# Regulation of Antimicrobial Peptide Activity via Tuning Deformation Fields by Membrane-Deforming Inclusions

**DOI:** 10.3390/ijms23010326

**Published:** 2021-12-28

**Authors:** Oleg V. Kondrashov, Sergey A. Akimov

**Affiliations:** Frumkin Institute of Physical Chemistry and Electrochemistry, Russian Academy of Sciences, 31/4 Leninskiy Prospekt, 119071 Moscow, Russia

**Keywords:** lipid membrane, antimicrobial peptide, gramicidin, theory of elasticity, partition function, Mayer cluster expansion, monomer-dimer equilibrium

## Abstract

Antimicrobial peptides (AMPs) are considered prospective antibiotics. Some AMPs fight bacteria via cooperative formation of pores in their plasma membranes. Most AMPs at their working concentrations can induce lysis of eukaryotic cells as well. Gramicidin A (gA) is a peptide, the transmembrane dimers of which form cation-selective channels in membranes. It is highly toxic for mammalians as being majorly hydrophobic gA incorporates and induces leakage of both bacterial and eukaryotic cell membranes. Both pore-forming AMPs and gA deform the membrane. Here we suggest a possible way to reduce the working concentrations of AMPs at the expense of application of highly-selective amplifiers of AMP activity in target membranes. The amplifiers should alter the deformation fields in the membrane in a way favoring the membrane-permeabilizing states. We developed the statistical model that allows describing the effect of membrane-deforming inclusions on the equilibrium between AMP monomers and cooperative membrane-permeabilizing structures. On the example of gA monomer-dimer equilibrium, the model predicts that amphipathic peptides and short transmembrane peptides playing the role of the membrane-deforming inclusions, even in low concentration can substantially increase the lifetime and average number of gA channels.

## 1. Introduction

Antimicrobial peptides (AMP) are considered as prospective candidates for the next generation of antibiotics; they are thought to be able to overcome multiple drug resistances of bacteria. In a variety of AMPs several (overlapping) classes can be distinguished: α-helical amphipathic peptides [1,2], β-hairpin peptides [3], channel-formers [4]. AMPs can fight bacteria via several mechanisms, among which the cell lysis or pore formation in the plasma membrane is relatively widespread [1,5,6,7]. The selectivity of AMPs towards bacteria is mainly based on negative electric charge of the outer leaflet of bacterial plasma membrane, as opposed to electrically neutral outer leaflets of membrane of eukaryotic cells. Thus, most of AMPs carry large positive electric charge to ensure strong binding of peptides to bacterial membranes [1,3,6].

Two types of pores are usually observed: (i) barrel-like, where the pore edge is formed exclusively by peptide molecules [8,9]; (ii) toroidal, where the edge is formed by both peptides and lipids [8,10,11]. The characteristic feature of amphipathic peptides having α-helical secondary structure is that their helix has one predominantly hydrophobic side surface, while the opposite side surface is mainly polar and charged. The ratio of hydrophobic/hydrophilic areas determines the so-called polar angle, which is considered to be responsible for the type of the pore formed by the peptides: larger polar angle yields barrel-like pores. AMPs having β-sheet secondary structure mostly form barrel-like pores of fixed stoichiometry, and, consequently, fixed pore diameter and conductivity [3].

The mechanisms of pore formation by membrane-bound peptides are still under debate. Experimentally, two configurations of AMPs are commonly observed: (i) a surface-bound S-configuration, in which the AMPs lie almost parallel to the membrane surface, having their hydrophobic parts incorporated into the lipid monolayer hydrophobic core; (ii) a membrane-spanning I-configuration, in which AMPs stand about perpendicularly to the membrane plane forming the edge of toroidal or barrel-like pore [8,10,12]. An intensive transformation from S to I-configuration leading to pore formation takes place at around certain so-called critical surface concentration of AMP, which usually weakly depends on particular AMP chemical structure, and for the most AMPs has the order of magnitude of about 40–100 lipids per peptide [1,8,10,13]. In the membrane-bound S-configurations AMPs incorporate their hydrophobic parts into the lipid monolayer, thus unavoidably inducing elastic deformations of the membrane [2,12,14,15,16]. The characteristic length of lateral spread of membrane deformations is about several nanometers [17,18,19]. When the membrane-deforming peptides are far separated, their induced deformations are independent and the corresponding elastic energies are additive. However, as the distance between the peptides decreases, the deformations overlap leading to effective lateral interaction between the peptides [19]. Deformations induced by a typical α-helical amphipathic peptide affect about 80 lipid molecules in the peptide vicinity [19,20] in both membrane leaflets; this value is pretty close to the AMP critical surface concentration. In other words, an intensive pore formation starts when, on the average, the whole membrane is covered by peptide-induced deformations. Substantially higher AMP concentrations usually lead to micellation of the membrane; in this case the AMP acts as a detergent [21]. Physical reasons of cooperative re-orientation of initially membrane-bound AMP molecules in the course of transformation to I-configuration, as well as the reasons of similarity of the critical concentration values among AMPs having different chemical structures are not yet resolved. In the work [22] the authors thoroughly analyzed approximately 16,000 different peptides 9–15 amino acid residues long, soluble in water and increasing the permeability of phospholipid membranes upon binding to them. As a result, 10 peptides with high rates of solubility and membrane permeabilization were selected. The analysis of the selected peptides did not reveal any unique features of the chemical structure. These extremely active peptides had no common sequence motifs, differed in peptide length and total electrical charge. However, the peptides had a similar secondary structure and core hydrophobicity. In an extensive review on membrane permeabilizing peptides [23], it is noted that there is no established single mechanism of action of such peptides, and that the membrane permeabilization is the result of a heterogeneous and dynamic ensemble of structural states of peptides that differ depending on a variety of experimental conditions. In this regard, there are practically no discrete active structures among the thousands of known AMPs, and there are no clear rules for determining the chemical structure of a peptide to achieve a given membrane activity. In this review, the authors propose not to analyze specific features of the chemical structure of peptides, but rather to consider the distribution of the totality of all membrane-permeabilizing peptides in the space of their mechanical action on lipid membranes.

Unfortunately, at the bulk concentrations of the AMP that are necessary to achieve the critical surface concentration, most AMPs effectively bind and permeabilize membranes of eukaryotic cells as well [1]. The AMP affinity towards a membrane of particular lipid composition is determined by peptide electric charge and by the tendency to hide the hydrophobic parts of the molecule inside the membrane hydrophobic core. The latter is achieved via partial incorporation of the peptide into the lipid monolayer, and it is the incorporation that is responsible for membrane deformations leading to peptide-peptide lateral interaction and cooperative pore formation. Thus, an effective AMP has to have a substantial hydrophobic part of the molecule. The hydrophobic interactions depend but weakly on the particular type of the membrane (bacterial or eukaryotic), and for this reason there should be always a fraction of AMPs that bind to eukaryotic cells, potentially leading to their permeabilization or even lysis. Such concomitant damage of eukaryotic cells substantially hinders the development and practical use of AMPs. A way to overcome this side effect is to develop highly effective AMPs to decrease their working concentrations. However, the critical AMP concentration is most probably determined by the characteristic lengths of membrane deformations, which should weakly depend on the particular type of the membrane (bacterial or eukaryotic). In this view, the use of synergistic combinations of antimicrobial agents seems to be a more reliable alternative [24,25,26]. The synergism may be achieved by action of two peptides on two consequent stages of the bacteria damage. For example, the first peptide can permeabilize the plasma membrane thus allowing the second peptide to reach its target inside the cell [25]. In the work [26] the synergistic effect was shown to be based on that the binding of the first peptide (PGLa) to the membrane substantially facilitated the binding of the second peptide (magainin 2) via relaxation of unfavorable membrane curvature strain.

A special class of AMPs is so-called channel-formers, a typical member of which is gramicidin A (gA) [27,28]. This pentadecapeptide is produced by *Bacillus brevis*. In membrane environment gA attains *β*^6.3^-helical conformation [29,30]. The longitudinal axis of the helix is tilted but slightly with respect to the membrane normal; such an orientation is maintained by three tryptophan amino acids at gA C-terminal [28,31,32]. Two gA monomers located in the opposing leaflets of the membrane can reversibly form a cation-selective ion channel via transmembrane head-to-head dimerization [27,29]. Normally, the conducting dimer is stabilized by six hydrogen bonds established between amino acids at N-terminal of two gA monomers [33]. Both dimerization and dissociation of the dimer are suggested to pass through the common state of so-called coaxial pair (denoted as P), in which two gA monomers from opposing lipid monolayers are located one on top of the other [34,35,36]. The coaxial pair is considered to correspond to the top of the energy barrier of the dimerization/dissociation process, whereas the states of two monomers or conducting dimer correspond to (local) minima of the free energy. In all three characteristic states (far-separated monomers, coaxial pair, conducting dimer) gA induces elastic deformations of the membrane [31,32,34,35,36]. In turn, elastic parameters of the membrane (thickness, spontaneous curvature, lateral tension, elastic rigidity) substantially affect the characteristics of the channel (lifetime and probability of formation) [37,38,39]. This allowed utilizing gramicidin as a molecular sensor of membrane elasticity [40,41,42]. Various membrane-deforming inclusions, like amphipathic peptides, are also expected to influence the lifetime and probability of formation of the conducting channel via deformation-mediated lateral interactions with gA monomers, dimers and coaxial pairs. The interactions may differently contribute to the energies of these states, thus modifying the height of the energy barriers of gA dimerization/dissociation. Recently, we have demonstrated that even gramicidin monomers themselves may serve as such membrane-deforming inclusions, and they can influence the characteristics of the channel via deformation-mediated lateral interactions with the dimer and coaxial pair [36]. Lateral dimers of gA may be formed via connection of two gA monomers located in the same membrane leaflet by a linker [43,44,45]. In a series of works [43,44,45] it is demonstrated that the lifetime of so-called tandem channels of gA formed from two lateral dimers located in opposing membrane leaflets, exceed the lifetime of the regular gA channel by about three orders of magnitude. This effect can be also quantitatively explained by strong membrane-mediated lateral interactions between two transmembrane dimers of gA that comprise the tandem channel [35]. The experimentally observed strong influence of membrane-mediated lateral interactions on gA channel characteristics makes gA a convenient tool to validate predictions derived based on the theory of elasticity of lipid membranes.

The practical use of gA as an antimicrobial agent is very limited because of its high toxicity. In highly-organized organisms, relatively hydrophobic gA molecules incorporate into cellular plasma membranes without any significant selectivity towards bacterial cells [46,47]. Monomer-dimer equilibrium of gA is strongly shifted towards monomers. If gA concentrations are sufficient to raise the permeability of bacterial membranes, the permeability of eukaryotic membranes will raise as well. At smaller concentrations, the average number of gA channels is too small to cause any significant antimicrobial effect.

Here we attempt to build a rational basis for development of specific molecules amplifying the membrane-permeabilizing activity of AMPs in the target (bacterial) membrane. The amplifiers selectively binding to bacterial membranes are supposed to allow reducing working concentrations of AMPs and simultaneously increase their selectivity. Permeabilization of the membrane via formation of barrel-like pore, toroidal pore or ion channel is accompanied by elastic deformations of the membrane, and these deformations contribute to characteristics of the membrane-permeabilizing structures. In other words, the average conductivity of the membrane should depend on the spatial structure of its deformations. Rigid membrane inclusions that purposefully modify the deformation fields of the membrane can thus potentially regulate the activity of AMPs. The application of the amplifiers would allow separating the requirements to AMPs in terms of high specificity and activity. With the use of amplifiers, AMPs may have a relatively low selectivity, and the amplifiers may not have the ability to permeabilize the membrane at all. With the simultaneous addition of AMP in a low (non-toxic for eukaryotic cells) concentration and the amplifiers of its activity, AMP can be incorporated into membranes of almost all cells. However, the permeabilization should occur exclusively in bacterial membranes due to the selective incorporation of the amplifiers of AMP activity into bacterial membranes. Due to the selective incorporation into bacterial membranes of both the AMP itself and the amplifier of its activity, the effective distribution coefficient between the membranes of bacterial and eukaryotic cells of such a combined antimicrobial drug is determined by the product of the distribution coefficients of its individual components (AMP and activity amplifier).

In a number of works devoted to investigation of the molecular mechanisms of AMP action, a small number of peptides simultaneously acting on the membrane are considered [14,16]. However, the permeabilization of membranes by AMPs at a certain surface concentration is a stochastic process, and the average membrane conductivity induced by AMPs is a statistical value. Thus, it seems reasonable to use statistical approaches for understanding and adequate description of AMP action mechanisms and for estimation of the membrane conductivity under certain experimental conditions. For definiteness, here we develop the statistical model for conductivity of the membrane induced by gramicidin A, although the consideration can be generalized for other types of AMPs inducing elastic deformations. As potential amplifiers of the gA activity, we consider single-pass transmembrane peptides, e.g., WALP or KALP [48,49,50], and short amphipathic peptides. Both type of peptides (denoted as I, inclusion) are supposed to carry a chemical group responsible for the selective binding to bacterial membranes. We demonstrate that even low concentrations of amphipathic peptides (e.g., 1 peptide per 2500 lipids) and extremely low concentrations of short transmembrane peptides (e.g., 1 peptide per 100,000 lipids) are sufficient to substantially (several times) alter the average characteristics of the gA channel ensemble (i.e., lifetime and number of conducting dimers).

## 2. Results

### 2.1. Algorithm of the Model Development

**Figure 1:**
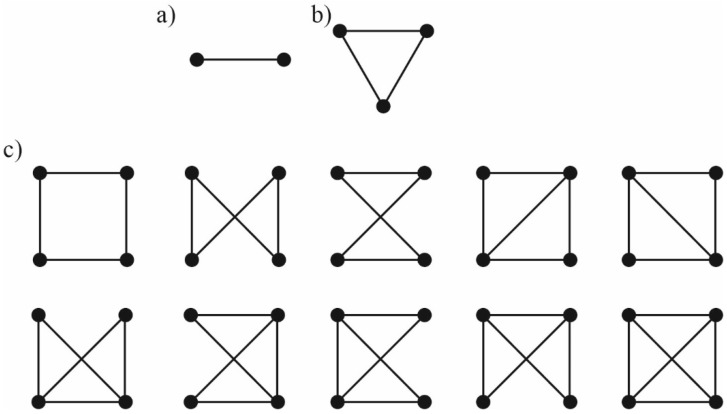
Irreducible diagrams for calculation of the partition function in the necessary order of approximation on particle concentrations. (**a**)—linear diagram for calculation of the partition function in the second order on concentrations; (**b**)—triangular diagram to calculate the third-order contributions to the partition function; (**c**)—the fourth order diagrams allowing obtaining the partition function in the fourth order on concentrations.

Consider the membrane with *N*_1_ monomers of gA in the upper monolayer (denoted as U), *N*_2_ monomers of gA in the lower monolayer (denoted as L), and *m* particles of the inclusion (denoted as I). At the moment, gA monomers from opposing membrane leaflets form *k* conducting dimers (denoted as D). The rate of transition of gA monomers between the leaflets is assumed to be very low, and thus the numbers of gA monomers in each leaflet *N*_1_, *N*_2_ are constant. First, we derive the partition function *Z*_0_ of the membrane with adsorbed gA molecules and incorporated inclusions. In the zeroth approximation, we consider the ideal system, where all possible membrane-mediated interactions of gA species and inclusions are absent. In order to account for the interactions of gA species and membrane inclusions (all particles with all ones), we utilize the Mayer cluster expansion [51,52,53]. In this approach, it is supposed that the configuration partition function of the non-ideal system can be expressed as a product of specific functions of pairwise interaction potentials; each function is averaged with respect to all possible positions of two interacting particles accounting for the Gibbs weight of the configurations. For gA monomers incorporated into two (upper and lower) leaflets of the membrane that are able to form conducting dimers, in the presence of inclusions, there are ten such pairwise interaction functions corresponding to the total number of ways to choose couples of particles from four types of particles present in the system: gA monomer in the upper monolayer (U), gA monomer in the lower monolayer (L), conducting dimer (D), inclusion I: U + U, U + L, L + L, U + D, L + D, U + I, L + I, D + D, I + I, D + I. In the limit of low concentrations, the pairwise functions are statistically independent and the average of their product can be substituted by the product of their average values. This substitution yields the first approximation of the partition function. Following van Kampen [52], we look for the higher-order corrections by means of correcting factors. In particular, the second correction should include three-particle correlations yielding the factors in the partition function in an amount equal to the number of ways to select three particles from four types of particles (U, L, D, I) present in the system; if we had only one kind of particles, there would be *N* × (*N* − 1) × (*N* − 2)/3! of such factors. Utilizing this approach, for known potential energy of particle pairwise interactions, one can calculate the coordination partition function in any desired order on concentrations. Generally, the factors for calculation of the partition function in the second order approximation on concentrations can be presented as the linear diagram (Figure 1a), third order—as the triangular diagrams (Figure 1b), fourth order—as square-like or fourth order diagrams shown in Figure 1c. In the diagrams, each vertex corresponds to the particle of the particular type, each edge corresponds to the function *f*_AB_, which characterizes the interaction of the particles A and B, standing in the corresponding vertices, *f*_AB_ = exp[–*w*_AB_/(*k_B_T*)] − 1, where *w*_AB_ is the pairwise interaction potential of particles A and B, *k_B_* is the Boltzmann constant, *T* is the absolute temperature. The interactions should be integrated over all possible configurations (positions) of the particles yielding the Mayer’s cluster integrals, and then should be summed over all possible types of particles. All illustrated diagrams are irreducible [51,52,53]. Utilizing the described approach, for known potential energy of particle pairwise interactions, one can calculate the coordination partition function in any desired order on concentrations. In the present work, we consider the partition function in the fourth order on concentrations.

The potential energy of the pairwise interaction of the particles was considered as the energy of elastic deformations of the membrane in the corresponding configuration of the particles. The elastic energy was calculated in the framework of the theory of elasticity of lipid membranes [20,34,35,36,54,55]. The gA monomer, dimer and membrane inclusion differed by the type of the boundary conditions they impose on the membrane deformations. The obtained potential energies allowed calculating numerically the Mayer’s integrals of the necessary order. The Mayer’s integrals were then used to obtain the partition functions of the system. The partition functions allowed calculating the dependences of the average lifetime of the gA channels as well as the average number of dimers in the membrane (i.e., the total membrane conductivity) on the concentrations of gA monomers and membrane inclusions (peripheral or transmembrane).

### 2.2. Pairwise Interaction Potentials

**Figure 2:**
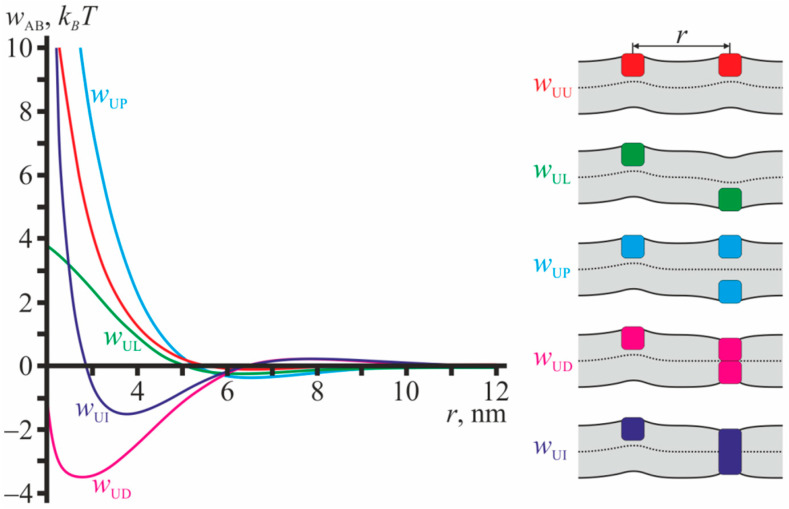
Profiles of pairwise interaction potentials for configurations involving gA monomers. Red curve—*w*_UU_(*r*); green curve—*w*_UL_(*r*); blue curve—*w*_UP_(*r*); magenta curve—*w*_UD_(*r*); dark blue curve—*w*_UI_(*r*) (for transmembrane inclusion). The configurations are schematically illustrated in the right-hand-side of the figure. The membrane is shown as light gray stripe; solid black lines correspond to surfaces of the upper and lower monolayers; dotted line corresponds to the monolayer interface; gA monomers are shown as squares; the transmembrane inclusion is shown as the rectangle. The colors of the particles in the right-hand-side of the figure correspond to the colors of the interaction potential curves *w*_AB_.

The profiles of pairwise interaction potentials calculated in the framework of the theory of elasticity of lipid membranes are illustrated in Figure 2 for configurations involving gA monomers. From Figure 2 it is seen that the interaction potentials with gA monomers are mostly repulsive except that for monomer-dimer (*w*_UD_) and monomer-transmembrane inclusion (*w*_UI_). The potentials decay rapidly with increasing distance between the particles, and at *r* ≈ 10 nm the interactions are negligibly weak.

All dependences *w*_AB_ illustrated in Figure 2 have global minima. The minima are relatively deep when gA monomers interact with transmembrane particles: for dimer, *w*_UD_, and transmembrane inclusion, *w*_UI_, the depths of the minima are ~3.5 *k_B_T* and ~1.5 *k_B_T*, respectively (Figure 2). The minima of the potentials that do not involve transmembrane particles are relatively shallow and their depths do not exceed about 0.5 *k_B_T* (curves *w*_UU_, *w*_UL_, *w*_UP_ in Figure 2). In all configurations considered in Figure 2, the monolayer interface is not flat as the particle configurations and their induced deformations do not possess mirror symmetry with respect to the plane of the unperturbed membrane.

**Figure 3:**
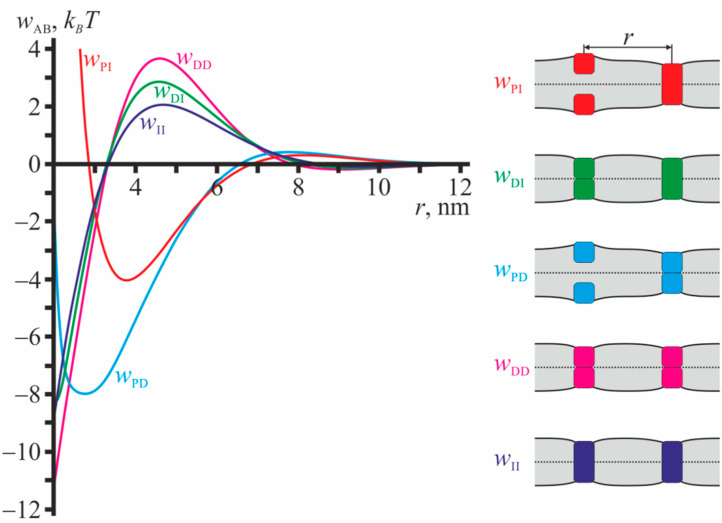
Profiles of pairwise interaction potentials for configurations that do not involve single gA monomers. Red curve—*w*_PI_(*r*); green curve—*w*_DI_(*r*); blue curve—*w*_PD_(*r*); magenta curve—*w*_DD_(*r*); dark blue curve—*w*_II_(*r*) (for transmembrane inclusion). The configurations are schematically illustrated in the right-hand-side of the figure. The membrane is shown as light gray stripe; solid black lines correspond to surfaces of the upper and lower monolayers; dotted line corresponds to the monolayer interface; gA monomers are shown as squares; the transmembrane inclusions are shown as rectangles. The colors of the particles in the right-hand-side of the figure correspond to the colors of the interaction potential curves *w*_AB_.

Figure 3 illustrates the profiles of pairwise interaction potentials for configurations that do not involve gA monomers. From Figure 3 it is seen that the interaction potentials in such configurations are mostly attractive. The potentials decay rapidly with increasing distance between the particles, and at *r* ≈ 10 nm the interactions are negligibly weak. All dependences *w*_AB_ illustrated in Figure 3 have global minima, and their depths are quite large: from ~4 *k_B_T* for *w*_PI_ up to ~11 *k_B_T* for *w*_DD_. All configurations illustrated in Figure 3 are mirror symmetric with respect to the plane of the unperturbed membrane; the monolayer interface is flat.

### 2.3. Influence of Lateral Interaction on the Equilibrium Number of Dimers

The pairwise interaction potentials presented in Figure 2 and Figure 3 were utilized to numerically calculate the Mayer’s cluster integrals of the necessary order. The Mayer’s integrals were then used to obtain the partition functions of the system. Some intermediate equations could not be solved analytically, and thus, to obtain the approximate solutions, we utilized the perturbation theory. The partition functions allowed obtaining the dependence of the dimer-monomer equilibrium constant on the concentration of gramicidin monomers, amphipathic membrane inclusions, and transmembrane inclusions in the membrane. This dependence leads to the shift of the equilibrium number of conducting dimers, *C*_D_, as compared to the case of absent lateral interactions, $C_{D}^{id}$. Below, we graphically illustrate the dependence of the ratio *C*_D_/$C_{D}^{id}$ = 1 + Δ*C*_D_/$C_{D}^{id}$ (see Equation (24)) on the concentration of gramicidin monomers, amphipathic membrane inclusions, and transmembrane inclusions. For simplicity, we consider the case *C*_U_ = *C*_L_ = *C*_1_ = *C*_2_ corresponding to equally distributed gramicidin monomers over the membrane leaflets. Besides, we analyzed the consequences of membrane-mediated lateral interactions on gA analogues with N-terminal valine replaced by glycine ([Gly1]gA) or tyrosine ([Tyr1]gA). These analogues have significantly reduced the monomer-dimer equilibrium constants *D*_0_, with monomer-dimer equilibrium being shifted towards monomers as compared to gA [36]. The values of monomer-dimer equilibrium constants (product of dimerization constant and dimer lifetime) determined in the work [36] for gA, [Gly1]gA, [Tyr1]gA are as follows: 25,812 nm^2^, 250.8 nm^2^, 52.5 nm^2^, respectively.

**Figure 4:**
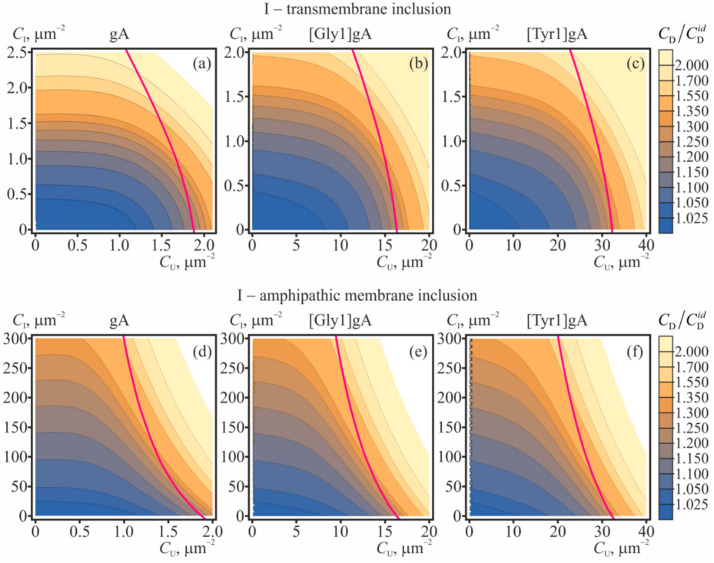
The dependence of the equilibrium concentration of conducting dimers on concentrations of gramicidin monomers and membrane inclusions: (**a**–**c**)—transmembrane inclusions; (**d**–**f**)—amphipathic membrane inclusions, incorporated into the top monolayer. The value *C*_D_/$C_{D}^{id}$ = 1 + Δ*C*_D_/$C_{D}^{id}$ is plotted for: (**a**,**d**)—gA, (**b**,**e**)—[Gly1]gA, (**c**,**f**)—[Tyr1]gA. The magenta curves bound the range of concentrations where the correction to the main approximation calculated within the perturbation theory reaches 20%; for larger *C*_U_, *C*_I_ concentrations the application of the perturbation theory is not strictly valid. The illustrated dependences of *C*_D_/$C_{D}^{id}$ were obtained based on Equation (24).

The dependences of the equilibrium concentration of conducting dimers on concentrations of gramicidin monomers and membrane inclusions (amphipathic or transmembrane) are shown in Figure 4 for gA, [Gly1]gA, and [Tyr1]gA. The magenta curves in Figure 4 bound the range of concentrations where the correction to the main approximation calculated within the perturbation theory reaches 20%. This value of the correction was arbitrary chosen to qualitatively mark the limits of applicability of the perturbation theory.

From Figure 4 it is seen that increasing concentrations of gramicidin monomers (*C*_U_ = *C*_L_), amphipathic membrane inclusions and transmembrane inclusions lead to increase of the ratio *C*_D_/$C_{D}^{id}$. The most pronounced growth of the number of the conducting dimers is induced by transmembrane inclusions (Figure 4a–c): the doubling of the conducting dimer number is provided by only 2.5 particles per 1 μm^2^. Although the amphipathic membrane inclusions cause appreciable increase of the *C*_D_/$C_{D}^{id}$ in substantially larger concentrations (e.g., hundreds of particles per 1 μm^2^, (Figure 4d–f)), practically, it is much easier to incorporate amphipathic peptides into the membrane as compared to transmembrane peptides.

### 2.4. Influence of Lateral Interaction on the Average Lifetime of Dimers

**Figure 5:**
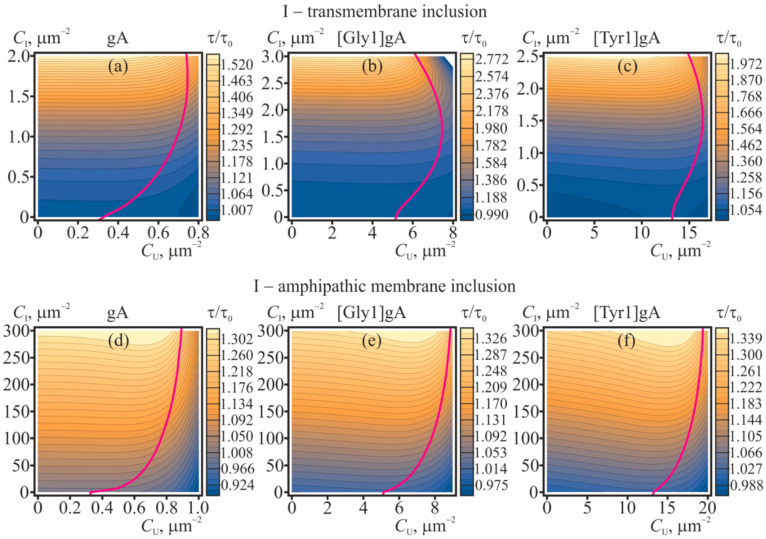
The dependence of the dimer lifetime (relative to its value in the “ideal” case) on concentrations of gramicidin monomers and membrane inclusions: (**a**–**c**)—transmembrane inclusions; (**d**–**f**)—amphipathic membrane inclusions, incorporated into the top monolayer. The value *τ*/*τ*_0_ is plotted for: (**a**,**d**)—gA; (**b**,**e**)—[Gly1]gA; (**c**,**f**)—[Tyr1]gA. The magenta curves bound the range of concentrations where the correction to the main approximation calculated within the perturbation theory reaches 20%; for larger *C*_U_, *C*_I_ concentrations the application of the perturbation theory is not strictly valid. The illustrated dependences of *τ*/*τ*_0_ were obtained based on Equation (29).

**Figure 6:**
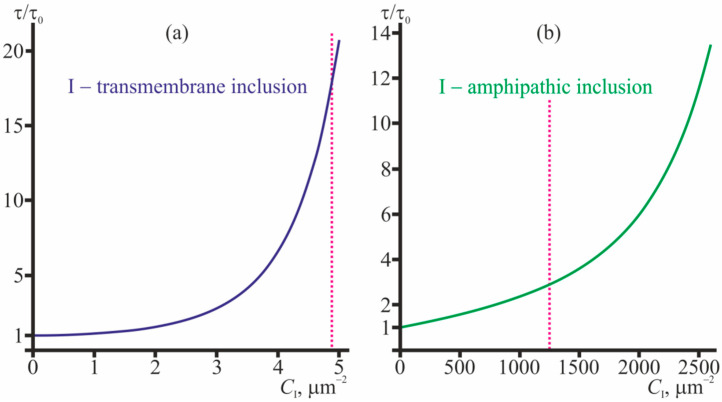
The dependence of the relative lifetime, *τ*/*τ*_0_, of the solitary conducting dimer on (**a**)—concentration of transmembrane inclusions; (**b**)—concentration of amphipathic membrane inclusions (incorporated into the top monolayer). The dependencies were obtained based on Equation (31). The magenta dotted lines bound the range of concentrations where the correction to the main approximation calculated within the perturbation theory reaches 20%; for larger *C*_I_ concentrations the application of the perturbation theory is not strictly valid.

The membrane-deforming inclusions influence the lifetime of the conducting dimers of gramicidin. The dissociation constant, which is the inverse lifetime of the dimer, is determined by the difference of the energies of the coaxial pair and the dimer [34,35,36]. Partially, these energies include contributions from membrane elastic deformations, which, in turn, can be modulated by membrane-deforming inclusions. In order to characterize the effect of membrane-mediated lateral interactions, we consider the ratio *τ*/*τ*_0_ of the average dimer lifetime, *τ*, and this value *τ*_0_ in the “ideal” case of absence of any lateral interactions. The dependences of *τ*/*τ*_0_ on concentrations of gramicidin monomers and membrane inclusions (amphipathic or transmembrane) are shown in Figure 5 for gA, [Gly1]gA, and [Tyr1]gA. The magenta curves in Figure 5 bound the range of concentrations where the interaction-related correction to the main “ideal” approximation calculated within the perturbation theory reaches 20%. This value of the correction was arbitrary chosen to qualitatively mark the limits of applicability of the perturbation theory. From Figure 5 it follows that increasing concentrations of amphipathic membrane inclusions and transmembrane inclusions lead to increase of the ratio *τ*/*τ*_0_. The most pronounced growth of the dimer lifetime is induced by transmembrane inclusions (Figure 5a–c): a 2–3 times increase of the lifetime is provided by only 2–3 particles per 1 μm^2^. Amphipathic membrane inclusions cause appreciable increase of the dimer lifetime in substantially larger concentrations—hundreds of particles per 1 μm^2^, (Figure 5d–f). The level curves in contour plots in Figure 5 are approximately parallel to the axis of gramicidin monomer concentration (*C*_U_) meaning that the dependence of *τ*/*τ*_0_ on *C*_U_ is relatively weak. In the limit *C*_U_ → 0 one can obtain the simple expression, Equation (31), for the dependence of the lifetime of the solitary dimer on the concentration of membrane inclusions. This dependence is illustrated in Figure 6 for transmembrane and amphipathic membrane inclusions.

From Figure 6 it is seen that the average lifetime of the solitary conducting dimer grows with increasing concentrations of transmembrane inclusions and amphipathic membrane inclusions. An about order-of-magnitude increase in *τ*/*τ*_0_ is provided by 4.5 transmembrane peptides or 2400 amphipathic peptides per 1 μm^2^.

## 3. Discussion

In the present study we considered the consequences that the membrane-mediated lateral interactions have on characteristics of the gramicidin ensemble and solitary dimers. For the analysis, we developed the statistical model that accounted for the interactions in the fourth order on concentrations of membrane-incorporated particles. The interactions were assumed to be mediated by elastic deformations of the membrane, induced by the membrane-incorporated particles. The profiles of the potential energy of pairwise interactions of the particles were calculated in the framework of theory of elasticity of lipid membranes [20,34,35,36,54,55]. Generally, the interaction involving gramicidin monomers are mostly repulsive (Figure 2), while the interactions involving transmembrane particles are mostly attractive (Figure 3). From the model it follows that gramicidin monomers themselves stimulate formation of conducting dimers excessive over the number of dimers would be in the “ideal” case of absent lateral interactions (Figure 4), while the average lifetime of the conducting dimer weakly depends on the monomer concentration (Figure 5). Along with gramicidin species (monomers, dimers, coaxial pairs), we considered “inclusions”—membrane-deforming peptides, either transmembrane or amphipathic (peripheral). Short transmembrane peptides were predicted to substantially increase both the number of conducting dimers and the average lifetime of the dimers in very low concentrations, of the order of several peptide molecules per 1 μm^2^ (Figure 4a–c, Figure 5a–c and Figure 6a). Amphipathic peptides alter the gramicidin characteristics less strongly: a substantial increase in the number of conducting dimers and the lifetime of the dimer requires concentrations of the order of hundreds-thousands of amphipathic peptides per 1 μm^2^ (Figure 4d–f, Figure 5d–f and Figure 6b). Both transmembrane inclusions and amphipathic membrane inclusions act as amplifiers of gramicidin activity, as they stimulate formation of conducting dimers and increase the average lifetime of the conducting state (Figure 4, Figure 5 and Figure 6). If these inclusions have higher affinity towards particular membranes, e.g., they preferentially incorporate into negatively charged bacterial membranes, the inclusions would amplify the gramicidin activity predominantly in these membranes. The high selectivity of the inclusions and their amplification of gramicidin activity should allow reducing substantially the working concentrations of the gramicidin (e.g., below the toxic level for eukaryotic cells) still preserving a strong antimicrobial effect. Although the amphipathic membrane inclusions cause appreciable increase of the *C*_D_/$C_{D}^{id}$ in substantially larger concentrations than the transmembrane inclusions do, practically, it is much easier to incorporate amphipathic peptides into the membrane as compared to transmembrane peptides. Amphipathic peptides themselves can cause formation of pores in the membrane. However, the critical concentrations of membrane disruption or lysis by amphipathic peptides such as magainin-2 are of the order of one peptide per 40–100 lipid molecules [1,8,10,13]. The typical concentration of 1000 amphipathic peptides per 1 μm^2^ that is sufficient to provide substantial amplification of the gramicidin activity (Figure 4d–f and Figure 6b) corresponds to approximately one amphipathic peptide per 2850 lipids, i.e., about two orders of magnitude smaller than the membrane lytic concentration.

The developed model is quite general; its possible applications are not limited to the case of gramicidin. The model may be utilized for analysis of amplifying or weakening effect of various membrane-deforming inclusions with respect to different types of AMPs. The only requirement is that at the sequential stages of the action, the AMP should induce different deformations of the membrane. For the example of gramicidin this means that the spatial distributions of elastic deformations of the membrane induced by gramicidin monomers, coaxial pair and dimer should be different. In the case of pore-forming AMPs, elastic deformations of the membrane can be induced by AMPs themselves, pores and membrane-incorporated inclusions. Accounting for the energy of the deformations, one can obtain the membrane-mediated interaction potentials, similar to those illustrated in Figure 2 and Figure 3. The potentials can be further used to calculate the configuration partition functions and, consequently, to analyze the modulation effect of membrane inclusions on the pore-forming activity of AMPs, following the formalism developed in the “Materials and Methods” section.

The considered mechanism of the purposeful change in the equilibrium constant of the dimerization reaction is of a general nature and is not specific for gramicidin. In particular, we expect that in multiple systems, where membrane-deforming particles (monomers) can form higher-order aggregates (dimers, trimers, etc.) an addition of deformation-inducing inclusions can change the equilibrium distribution of the aggregate numbers. In such reactions, the inclusions do not play a role of a catalyst, as catalysts do not influence the chemical equilibrium. The inclusions strongly favor or stabilize a particular state or aggregate (e.g., the dimer in the case of gramicidin, Figure 3) thus shifting the equilibrium towards that state. This is rather similar to the action of the excessive external pressure on the equilibrium A + B ⇔ AB, where the volume of (A + B) reagents exceeds substantially the volume of AB product. In this case, a higher pressure favors smaller volume of the system, thus shifting the equilibrium to the right, towards AB product. The analogous effect should have an increased temperature if the direct reaction is endothermic, while the opposite reaction is exothermic. Commonly, in three-dimensional systems, the equilibrium constants weakly depend on the concentrations of the reagents. E.g., the Debye shielding works in solution; besides, when two oppositely charged particles combine, their charges cancel each other out and the combined particle (dimer) can interact with neighboring particles only via dipole and other interactions that are weaker than Coulomb interactions. In this sense, membranes are specific systems that allow particles to interact via relatively long-range deformations.

Fundamentally, the membrane with incorporated peptides can be considered as the system of many electric charges that mutually electrostatically interact. These interactions should alter when the membrane is disturbed by the inclusions, and thus the electrostatic-based interactions should contribute to the total energy of the system. However, when the membrane inclusion deforms the membrane, the bilayer thickness changes, the monolayer surfaces become curved, the monolayer surface can locally compress or stretch, and the axes of lipid molecules can deviate from the normal to the monolayer surface (i.e., the molecules can tilt). Let’s focus on the bending (curving) of the monolayer. Denote the local curvature of the monolayer surface as *J*. Assuming *J* small, one can take Taylor series of the free energy *F* on the small deviation of the curvature from zero: $F\left(J\right)\approx F\left(J=0\right)+{\frac{\partial F}{\partial J}|}_{J=0}J+\frac{1}{2}{\frac{\partial^{2}F}{\partial J^{2}}|}_{J=0}J^{2}$. Here, the first term is constant. The second term is zero, as we take Taylor series in the vicinity of the equilibrium state, where the free energy is minimal. In the third term, the combination ${\frac{\partial^{2}F}{\partial J^{2}}|}_{J=0}$ is referred to as the bending modulus (denoted by *B*, see Materials and Methods section). In our model, we utilized the values of the elastic moduli that were determined experimentally. This means that our used value of the bending modulus includes (within the required accuracy) all possible contributions from all fundamental physical forces (electrostatic, van der Waals, entropy-based, etc.) that arise upon deviation of the monolayer surface curvature from zero. This is due to the fact that all these forces and all these contributions acted when the modulus was experimentally determined and contributed to its measured value. The same can be repeated for the other deformation modes (lateral stretching, tilt, etc.). Thus, in our calculations, the contribution from, e.g., electrostatic and van der Waals interactions acting in the system of many charges are accounted for within the required accuracy via the experimentally determined values of the elastic moduli.

For gramicidin A, the elastic approach for the description of monomer-dimer equilibrium was originally developed in 1986 [56] and then was modified in around 2000 to account for the effects of monolayer spontaneous curvature [57,58]. The general statement of the model is that the energy of elastic deformations arising in the vicinity of gA dimer depends quadratically on the hydrophobic mismatch (*d*_0_ − *l*) between the length of the gA dimer (*l*) and bilayer thickness (*d*_0_). Thus, the model is based on linear elastic theory (on the Hook’s law), and is principally limited by the case of small deformations, i.e., (*d*_0_ − *l*) should be much smaller than *d*_0_ and *l*: 2(*d*_0_ − *l*)/(*d*_0_ + *l*) << 1. Note, that the particular limits of the linear theory applicability (i.e., the quantitative meaning of the sign “<<”) cannot be determined within this theory; it can be only verified experimentally or, e.g., in molecular dynamics (MD). The fundamental flaw of the original hydrophobic mismatch-based elastic model was that it considered only the deformations induced by the gA dimer and assumed that neither gA monomer nor the gA coaxial pair (which we consider as the transition state in monomer-dimer reaction) deform the membrane. However, if the gA monomer length along the normal to the membrane surface is smaller than the monolayer thickness, elastic deformations should unavoidably appear in the gA monomer vicinity. As the gA monomer cannot impose boundary conditions onto the monolayer (bilayer) thickness, these deformations principally cannot be accounted for in the framework of hydrophobic mismatch-based models. This means, that the activation barriers of the dimerization and dissociation reactions of gramicidin are calculated not exactly correctly in such models, as the energy of deformations in the transition state and the energy of deformations induced by gA monomers are not taken into account. Thus, the single order parameter (bilayer thickness) introduced in the mismatch-based models is not sufficient to account for the deformations arising in the gA monomer vicinity. In our considerations, we utilize the additional order parameter, characterizing the average orientation of lipid molecules—so-called, director. The gA monomer was assumed to impose the boundary condition onto the director (Equation (38)) and this leaded to deformations of the membrane in the gA monomer vicinity.

Of note, the membrane-deforming inclusions alter the average energy of gramicidin monomers in the membrane. This may lead to shift of equilibrium distribution of monomers between the membrane and water phase. In this case, the actual concentrations of gramicidin monomers in the membrane should stand in the equations. Obviously, numerous fundamental physical forces act at the peptide-lipid boundary. In our model these interaction (electrostatic, van der Waals, entropy-related, etc.) determine the boundary conditions imposed on the parameters that describe the state of the lipid monolayer (director, thickness, etc.). The corresponding energy of interactions of peptide with the adjacent lipids yields a constant contribution. In our consideration we operate with interaction potentials that are normalized in a way to tend to zero as the distance between the peptides increases (Figure 2 and Figure 3), and thus any constant contributions do not influence our model and its results.

It should be noted that the obtained dependences (Figure 4, Figure 5 and Figure 6) are rather qualitative, since when calculating the interaction potentials, we used the boundary conditions derived from simple geometric considerations. The boundary conditions can be subjected to uncertainties, which can alter the quantitative values derived from the diagrams (Figure 4, Figure 5 and Figure 6). However, our elastic model was successfully utilized in several works, and provided the results that were in quantitative (or, at least, semi-quantitative) agreement with the numerous experimental data and the data obtained in MD, including: the dependence of gramicidin A dimer dissociation probability on the lateral tension of the membrane; dependence of the probability of dimer formation on the lipid membrane thickness; dependence of the dimer lifetime on the spontaneous curvature of monolayers; the interaction energy profile of two gA dimers; the radial distribution of bilayer thickness around the gA dimer; the lifetime of the tandem gA channels; the characteristic time of flicker conductance of the tandem gA channels; the dependence of the characteristic time of gramicidin monomer-dimer equilibration on the gramicidin concentration, both normal dependence for gA, and anomalous dependence observed for gA derivatives [Gly1]gA and [Tyr1]gA [34,35,36]. Thus, the utilized parameters of the model and the model itself can be considered as reliable.

It is further worth noting that we used the large area limit, i.e., we assumed that although the concentrations of all particles are relatively low, the total number of the particles is still large that allow utilizing the statistical approach for the ensemble description. Practically, in the membrane of a relatively small area, the range of concentrations considered in our model can correspond to just a few particles. In this case, it is incorrect to apply the statistical description of the dimer-monomer equilibrium to obtain the average number of conducting dimers (Figure 4). However, it is still correct to use the developed model to describe the effect of membrane inclusions and gramicidin monomers on the lifetime of a solitary dimer (Figure 6). Plasma membranes of both eukaryotic and bacterial cells may comprise a large number of membrane-deforming species (e.g., transmembrane and peripheral proteins, protein-lipid domains, etc.), which can take part in the modulation of the equilibrium between AMP monomers and cooperative membrane-permeabilizing structures. Thus, the introduced membrane-deforming inclusions would regulate the already modulated equilibrium. However, due to the high selectivity of the amplifiers towards the bacterial cells, they should nevertheless predominantly enhance the permeability of bacterial membranes.

## 4. Materials and Methods

### 4.1. General Expressions for Partition Functions

We develop the statistical model of gA ensemble in the presence of membrane-deforming inclusions in the following way. Consider the membrane with *N*_1_ monomers of gA in the upper monolayer (denoted as U), *N*_2_ monomers of gA in the lower monolayer (denoted as L), and *m* particles of the inclusion. At the moment, gA monomers from opposing membrane leaflet form *k* conducting dimers (denoted as D). The rate of transition of gA monomers between the leaflets is assumed to be very low, and thus the numbers of gA monomers in each leaflet *N*_1_, *N*_2_ are constant. The partition function of such system can be expressed as:

(1) $$Z=\frac{1}{N_{1}!N_{2}!}\sum_{k=0}^{\min(N_{1},N_{2})}\left(\frac{N_{1}!N_{2}!}{\left(N_{1}-k\right)!\left(N_{2}-k\right)!k!}\right)\cdot Z_{0}\left(N_{1},N_{2},k,m\right),$$

where the summation is performed over the all possible numbers *k* of conducting dimers; the factor in the brackets is the number of ways to assemble *k* dimers from *N*_1_, *N*_2_ monomers of gA; *Z*_0_(*N*_1_, *N*_2_, *k*, *m*) is the partition function of this system with fixed number of conducting dimers. In the zeroth approximation, we consider the ideal system, where all possible membrane-mediated interactions of gA species and inclusions are absent. In this approximation, the partition function can be written as follows:

(2) $$\begin{array}{cc} Z_{0}\left(N_{1},N_{2},k,m\right) & =e^{\frac{-kW_{D}}{T}}{\left(\frac{\int \int_{S}d^{2}r_{U}e^{-\frac{Mv_{U}^{2}}{2T}}d^{2}v_{U}}{{\left(2\pi \hbar \right)}^{2}}\right)}^{N_{1}-k}{\left(\frac{\int \int_{S}d^{2}r_{L}e^{-\frac{Mv_{L}^{2}}{2T}}d^{2}v_{L}}{{\left(2\pi \hbar \right)}^{2}}\right)}^{N_{2}-k}\\ & \times {\left(\frac{\int \int_{S}d^{2}r_{D}e^{-\frac{2Mv_{D}^{2}}{2T}}d^{2}v_{D}}{{\left(2\pi \hbar \right)}^{2}}\right)}^{k}{\left(\frac{\int \int_{S}d^{2}r_{I}e^{-\frac{M_{I}v_{I}^{2}}{2T}}d^{2}v_{I}}{{\left(2\pi \hbar \right)}^{2}}\right)}^{m}, \end{array}$$

where ℏ is the Planck’s constant; the temperature *T* is measured in thermal energy units; the first exponent is the Boltzmann factor (*W*_D_ is the energy of single dimer); four factors in the brackets determine the phase space accessible by U, L, D, and I, respectively. These factors are proportional to *TS*/*M* or *TS*/*M*_I_, where *S* is the area of the membrane, *M* and *M*_I_ are the effective masses of gA monomer or inclusion, respectively. The effective mass of the dimer is considered as a doubled effective mass of gA monomer; the effective mass is the actual mass of the peptide particle and the mass of lipid molecules involved in the peptide particle motion. In Equation (2) we omitted the internal partition functions of the particles (rotational, oscillatory, etc.) as they do not influence the results obtained. We introduce the function:

(3) $$Q\left(N_{1},N_{2},k,m\right)=\int \exp[-\frac{W}{T}]\prod_{i=1}^{N_{1}-k}dr_{U,i}\prod_{j=1}^{N_{2}-k}dr_{L,j}\prod_{l=1}^{k}dr_{D,l}\prod_{t=1}^{m}dr_{I,t},$$

where *W* is the energy of particular configuration of the system that depends on coordinates of all particles in the membrane; the integration is performed over all possible positions of the particles. In the case of non-interacting particles:

(4) $$Q\left(N_{1},N_{2},k,m\right)\approx Q^{\left(0\right)}\left(N_{1},N_{2},k,m\right)=S^{N_{1}-k+N_{2}-k+k+m}=S^{N_{1}+N_{2}-k+m}$$

(*S* is the area of the membrane). Utilizing the function *Q*, the partition function Equation (2) can be rewritten as:

(5) $$\begin{array}{cc} Z_{0}\left(N_{1},N_{2},k,m\right) & =e^{\frac{-kW_{D}}{T}}{\left(\frac{\int e^{-\frac{Mv_{U}^{2}}{2T}}d^{2}v_{U}}{{\left(2\pi \hbar \right)}^{2}}\right)}^{N_{1}-k}{\left(\frac{\int e^{-\frac{Mv_{L}^{2}}{2T}}d^{2}v_{L}}{{\left(2\pi \hbar \right)}^{2}}\right)}^{N_{2}-k}\\ & \times {\left(\frac{\int e^{-\frac{2Mv_{D}^{2}}{2T}}d^{2}v_{D}}{{\left(2\pi \hbar \right)}^{2}}\right)}^{k}{\left(\frac{\int e^{-\frac{M_{I}v_{I}^{2}}{2T}}d^{2}v_{I}}{{\left(2\pi \hbar \right)}^{2}}\right)}^{m}\cdot Q\left(N_{1},N_{2},k,m\right). \end{array}$$

In order to describe the dependence of gA monomer-dimer equilibrium and the gA channel lifetime on gA monomer concentrations in the presence of inclusions, one should calculate the function *Q* taking into account the membrane-mediated lateral interactions of dimers, monomers, and inclusions. In order to account for the interactions of gA species and membrane inclusions (all particles with all ones), we utilize the Mayer cluster expansion [51,52,53] in up to the fourth order on particle concentrations, considering the interactions as pairwise. In this approach, it is supposed that the configuration partition function of the non-ideal system can be expressed as:

(6) $$Q\left(N_{1},N_{2},k,m\right)=S^{N_{1}-k+N_{2}-k+k+m}\bar{\varphi_{12}\varphi_{13}\ldots \varphi_{N-1,N}},$$

where *φ_ij_* = exp(–*w_ij_*/*T*); *w_ij_* are pairwise interaction potentials of particles *i* and *j*; *N* is the total number of particles; the overline here and below denotes averaging with respect to all possible positions of the interacting particles. It can be explicitly demonstrated that *φ*_12_ and *φ*_13_ as well as *φ*_12_ and *φ*_34_ are statistically independent, and thus $\bar{\varphi_{12}\varphi_{13}}=\bar{\varphi_{12}}\cdot \bar{\varphi_{13}}$, $\bar{\varphi_{12}\varphi_{34}}=\bar{\varphi_{12}}\cdot \bar{\varphi_{34}}$. However, generally, $\bar{\varphi_{12}\varphi_{13}\varphi_{23}}\neq \bar{\varphi_{12}}\cdot \bar{\varphi_{13}}\cdot \bar{\varphi_{23}}$. The relation $\bar{\varphi_{12}\varphi_{13}\varphi_{23}}=\bar{\varphi_{12}}\cdot \bar{\varphi_{13}}\cdot \bar{\varphi_{23}}$ can be used as the first approximation in the case of low concentration of the particles, as in this case the configurations when all three functions *φ_ij_* differ from unity are relatively rare. Applying this approach, we can express the function *Q* in the first approximation as follows:

(7) $$\frac{Q^{\left(1\right)}\left(N_{1},N_{2},k,m\right)}{S^{N_{1}-k+N_{2}-k+k+m}}={\left(\bar{\varphi_{\mathrm{UU}}}\right)}^{\frac{1}{2}N_{U}\left(N_{U}-1\right)}{\left(\bar{\varphi_{\mathrm{LL}}}\right)}^{\frac{1}{2}N_{L}\left(N_{L}-1\right)}{\left(\bar{\varphi_{\mathrm{UL}}}\right)}^{N_{U}N_{L}}\ldots ,$$

where *φ*_AB_ = exp(–*w*_AB_/*T*) is the function corresponding to interaction of particles A and B. For gA monomers incorporated into two (upper and lower) leaflets of the membrane that are able to form conducting dimers, in the presence of membrane inclusions, there are ten such pairwise interaction functions corresponding to the total number of ways to choose couples of particles from four types of particles present in the system: gA monomer in the upper monolayer, gA monomer in the lower monolayer, conducting dimer, inclusion: U + U, U + L, L + L, U + D, L + D, U + I, L + I, D + D, I + I, D + I. In the limit of low concentrations, the pairwise functions are statistically independent and the average of their product can be substituted by the product of their average values. In the limiting case of large system (*S* → +∞, *N* → +∞, *N*/*S* = *C* = const) the expression Equation (7) can be rewritten as:

(8) $$\frac{Q^{\left(1\right)}\left(N_{1},N_{2},k,m\right)}{S^{N_{1}-k+N_{2}-k+k+m}}=\exp\left(\frac{N_{U}^{2}}{2S}\beta_{1,\mathrm{UU}}+\frac{N_{L}^{2}}{2S}\beta_{1,\mathrm{LL}}+\frac{N_{U}N_{L}}{S}\beta_{1,\mathrm{UL}}+\ldots \right),$$

where $\beta_{1,\mathrm{AB}}=\int \left(\varphi_{\mathrm{AB}}-1\right)dr_{\mathrm{AB}}$ is the Mayer’s first irreducible cluster integral [53], **r**_AB_ = **r**_A_ − **r**_B_ is the vector directed from the particle B to the particle A. The power of the exponent in Equation (8) includes 10 terms, each of which corresponds to the certain pair of interacting particles.

Following van Kampen [52], we look for the higher-order corrections by means of correcting factors. In particular, the second correction should include three-particle correlations yielding the factors in the partition function in an amount equal to the number of ways to select three particles from the four types of particles (U, L, D, I) present in the system; if we had only one kind of particles, there would be *N* × (*N* − 1)×(*N* − 2)/3! of such factors. Introducing the notation *φ*_AB_ = 1+ *f*_AB_, within the requited accuracy one can obtain:

(9) $$\begin{array}{l} \frac{\bar{\varphi_{12}\varphi_{13}\varphi_{23}}}{\bar{\varphi_{12}}\cdot \bar{\varphi_{13}}\cdot \bar{\varphi_{23}}}=\left(1+\frac{\bar{f_{12}f_{13}f_{23}}}{S^{2}}+O\left(\frac{1}{S^{3}}\right)\right),\\ \frac{Q^{\left(2\right)}\left(N_{1},\;N_{2},\;k,\;m\right)}{Q^{\left(1\right)}\left(N_{1},\;N_{2},\;k,\;m\right)}=\exp\left[\sum_{A,B,C}\frac{C_{\mathrm{ABC}}}{S^{2}}\bar{f_{\mathrm{AB}}f_{\mathrm{AC}}f_{\mathrm{BC}}}\right], \end{array}$$

where *C*_ABC_ is the number of ways to choose particles of types A, B, or C from the total number of particle types present in the system; the summation is performed over all possible triplets of the particles. In particular, for gA monomers in the upper leaflet, *C*_UUU_ = *N*_U_(*N*_U_ − 1)(*N*_U_ − 2)/3!; the number of ways to choose two gA monomers in the upper leaflet and the dimer is *C*_UUD_ = *N*_U_(*N*_U_ − 1)*k*/(2!·1!); the number of ways to choose gA monomer in the upper leaflet, gA monomer in the lower leaflet and the dimer is given by *C*_ULD_ = *N*_U_*N*_L_*k*/(1!·1!·1!), etc.

In order to obtain the next correcting factor, one should consider all possible groups of four particles and calculate the factor $\frac{\bar{\varphi_{12}\varphi_{13}\varphi_{23}\varphi_{14}\varphi_{24}\varphi_{34}}}{D}$ [52], where *D* is the approximation of the value $\bar{\varphi_{12}\varphi_{13}\varphi_{23}\varphi_{14}\varphi_{24}\varphi_{34}}$ obtained on the previous iteration. Generally, the factors for calculation of the partition function in the second order approximation on concentrations can be presented as the linear diagram (Figure 1a), third order—as the triangular diagrams (Figure 1b), fourth order—as fourth order diagrams shown in Figure 1c. In all diagrams, each vertex corresponds to the particle of the particular type, each edge corresponds to the function *f*. The interactions should be integrated over all possible configurations (positions) of the particles, and then should be summed over all possible types of particles (analogously to Equation (9)). All illustrated diagrams are irreducible [51,52,53]. Utilizing the described approach, for known potential energy of particle interaction, one can calculate the coordination partition function in any desired order on concentrations. We consider the partition function in the fourth order on concentrations. In this case, the expression for *Q*(*N*_1_, *N*_2_, *k*, *m*) can be presented as:

(10) $$Q\left(N_{1},\;N_{2},\;k,\;m\right)=S^{N_{1}+N_{2}-k+m}\exp\left[P\left(\frac{N_{U}}{S},\frac{N_{L}}{S},\frac{k}{S},\frac{m}{S}\right)\right],$$

where *P* is the polynomial (of the fourth order in our case) depending on the concentrations of particles of different types; the coefficients of *P* are determined by the diagrams illustrated in the Figure 1. Explicitly, the polynomial *P* can be written as follows:

(11) $$\begin{array}{cc} P & =[\beta_{1,\mathrm{UU}}\frac{{\left(N_{1}-k\right)}^{2}}{2!S}+\beta_{1,\mathrm{LL}}\frac{{\left(N_{2}-k\right)}^{2}}{2!S}+\beta_{1,\mathrm{UL}}\frac{\left(N_{1}-k\right)\left(N_{2}-k\right)}{S}+\beta_{1,\mathrm{UD}}\frac{\left(N_{1}-k\right)k}{S}\\ & +\beta_{1,\mathrm{LD}}\frac{\left(N_{2}-k\right)k}{S}+\beta_{1,\mathrm{UI}}\frac{\left(N_{1}-k\right)m}{S}+\beta_{1,\mathrm{LI}}\frac{\left(N_{2}-k\right)m}{S}+\beta_{1,\mathrm{DD}}\frac{k^{2}}{2!S}+\beta_{1,\mathrm{DI}}\frac{km}{S}+\beta_{1,\mathrm{II}}\frac{m^{2}}{2!S}]\\ & +[\beta_{2,\mathrm{UUU}}\frac{{\left(N_{1}-k\right)}^{3}}{3!S^{2}}+\beta_{2,\mathrm{LLL}}\frac{{\left(N_{2}-k\right)}^{3}}{3!S^{2}}+\beta_{2,\mathrm{UUL}}\frac{{\left(N_{1}-k\right)}^{2}\left(N_{2}-k\right)}{2!S^{2}}+\beta_{2,\mathrm{ULL}}\frac{\left(N_{1}-k\right){\left(N_{2}-k\right)}^{2}}{2!S^{2}}\\ & +\beta_{2,\mathrm{UUD}}\frac{{\left(N_{1}-k\right)}^{2}k}{2!S^{2}}+\beta_{2,\mathrm{LLD}}\frac{{\left(N_{2}-k\right)}^{2}k}{2!S^{2}}+\beta_{2,\mathrm{ULD}}\frac{\left(N_{1}-k\right)\left(N_{2}-k\right)k}{S^{2}}+\beta_{2,\mathrm{UUI}}\frac{{\left(N_{1}-k\right)}^{2}m}{2!S^{2}}\\ & +\beta_{2,\mathrm{LLI}}\frac{{\left(N_{2}-k\right)}^{2}m}{2!S^{2}}+\beta_{2,\mathrm{ULI}}\frac{\left(N_{1}-k\right)\left(N_{2}-k\right)m}{2!S^{2}}+\beta_{2,\mathrm{UDD}}\frac{\left(N_{1}-k\right)k^{2}}{2!S^{2}}+\beta_{2,\mathrm{LDD}}\frac{\left(N_{2}-k\right)k^{2}}{2!S^{2}}\\ & +\beta_{2,\mathrm{UDI}}\frac{\left(N_{1}-k\right)km}{S^{2}}+\beta_{2,\mathrm{LDI}}\frac{\left(N_{2}-k\right)km}{S^{2}}+\beta_{2,\mathrm{UII}}\frac{\left(N_{1}-k\right)m^{2}}{2!S^{2}}+\beta_{2,\mathrm{LII}}\frac{\left(N_{1}-k\right)m^{2}}{2!S^{2}}\\ & +\beta_{2,\mathrm{DII}}\frac{km^{2}}{2!S^{2}}+\beta_{2,\mathrm{DDI}}\frac{k^{2}m}{2!S^{2}}+\beta_{2,\mathrm{DDD}}\frac{k^{3}}{3!S^{2}}+\beta_{2,\mathrm{III}}\frac{m^{3}}{3!S^{2}}]\\ & +[\beta_{3,\mathrm{UUUU}}\frac{{\left(N_{1}-k\right)}^{4}}{4!S^{3}}+\beta_{3,\mathrm{LLLL}}\frac{{\left(N_{2}-k\right)}^{4}}{4!S^{3}}+\beta_{3,\mathrm{UUUL}}\frac{{\left(N_{1}-k\right)}^{3}\left(N_{2}-k\right)}{3!S^{3}}\\ & +\beta_{3,\mathrm{ULLL}}\frac{\left(N_{1}-k\right){\left(N_{2}-k\right)}^{3}}{3!S^{3}}+\beta_{3,\mathrm{UULL}}\frac{{\left(N_{1}-k\right)}^{2}{\left(N_{2}-k\right)}^{2}}{2!2!S^{3}}+\beta_{3,\mathrm{UUUD}}\frac{{\left(N_{1}-k\right)}^{3}k}{3!S^{3}}\\ & +\beta_{3,\mathrm{LLLD}}\frac{{\left(N_{2}-k\right)}^{3}k}{3!S^{3}}+\beta_{3,\mathrm{UULD}}\frac{{\left(N_{1}-k\right)}^{2}\left(N_{2}-k\right)k}{2!S^{3}}+\beta_{3,\mathrm{ULLD}}\frac{\left(N_{1}-k\right){\left(N_{2}-k\right)}^{2}k}{2!S^{3}}\\ & +\beta_{3,\mathrm{UUUI}}\frac{{\left(N_{1}-k\right)}^{3}m}{3!S^{3}}+\beta_{3,\mathrm{LLLI}}\frac{{\left(N_{2}-k\right)}^{3}m}{3!S^{3}}+\beta_{3,\mathrm{UULI}}\frac{{\left(N_{1}-k\right)}^{2}\left(N_{2}-k\right)m}{2!S^{3}}\\ & +\beta_{3,\mathrm{ULLI}}\frac{\left(N_{1}-k\right){\left(N_{2}-k\right)}^{2}m}{2!S^{3}}+\beta_{3,\mathrm{UUDI}}\frac{{\left(N_{1}-k\right)}^{2}km}{2!S^{3}}+\beta_{3,\mathrm{LLDI}}\frac{{\left(N_{2}-k\right)}^{2}km}{2!S^{3}}\\ & +\beta_{3,\mathrm{ULDI}}\frac{\left(N_{1}-k\right)\left(N_{2}-k\right)km}{S^{3}}+\beta_{3,\mathrm{UUII}}\frac{{\left(N_{1}-k\right)}^{2}m^{2}}{2!2!S^{3}}+\beta_{3,\mathrm{LLII}}\frac{{\left(N_{2}-k\right)}^{2}m^{2}}{2!2!S^{3}}\\ & +\beta_{3,\mathrm{ULII}}\frac{\left(N_{1}-k\right)\left(N_{2}-k\right)m^{2}}{2!S^{3}}+\beta_{3,\mathrm{UUDD}}\frac{{\left(N_{1}-k\right)}^{2}k^{2}}{2!2!S^{3}}+\beta_{3,\mathrm{LLDD}}\frac{{\left(N_{2}-k\right)}^{2}k^{2}}{2!2!S^{3}}\\ & +\beta_{3,\mathrm{ULDD}}\frac{\left(N_{1}-k\right)\left(N_{2}-k\right)k^{2}}{2!S^{3}}+\beta_{3,\mathrm{UDDI}}\frac{\left(N_{1}-k\right)k^{2}m}{2!S^{3}}+\beta_{3,\mathrm{LDDI}}\frac{\left(N_{2}-k\right)k^{2}m}{2!S^{3}}\\ & +\beta_{3,\mathrm{UDII}}\frac{\left(N_{1}-k\right)km^{2}}{2!S^{3}}+\beta_{3,\mathrm{LDII}}\frac{\left(N_{2}-k\right)km^{2}}{2!S^{3}}+\beta_{3,\mathrm{DIII}}\frac{km^{3}}{3!S^{3}}+\beta_{3,\mathrm{DDII}}\frac{k^{2}m^{2}}{2!2!S^{3}}+\beta_{3,\mathrm{DDDI}}\frac{k^{3}m}{3!S^{3}}\\ & +\beta_{3,\mathrm{UDDD}}\frac{\left(N_{1}-k\right)k^{3}}{3!S^{3}}+\beta_{3,\mathrm{LDDD}}\frac{\left(N_{2}-k\right)k^{3}}{3!S^{3}}+\beta_{3,\mathrm{UIII}}\frac{\left(N_{1}-k\right)m^{3}}{3!S^{3}}\\ & +\beta_{3,\mathrm{LIII}}\frac{\left(N_{2}-k\right)m^{3}}{3!S^{3}}+\beta_{3,\mathrm{DDDD}}\frac{k^{4}}{4!S^{3}}+\beta_{3,\mathrm{IIII}}\frac{m^{4}}{4!S^{3}}]. \end{array}$$

In this expression, the first, second and third square brackets correspond to linear, triangular and fourth order diagrams, respectively (Figure 1); $\beta_{2,\mathrm{ABC}}=\frac{1}{S}\int \int \int \left(e^{-\frac{W_{\mathrm{AB}}}{T}}-1\right)\left(e^{-\frac{W_{\mathrm{BC}}}{T}}-1\right)\left(e^{-\frac{W_{\mathrm{AC}}}{T}}-1\right)dr_{A}dr_{B}dr_{C}$; the coefficients *β*_3,ABCE_ are calculated in the analogous way.

### 4.2. Equilibrium between gA Monomers and Dimers in the Case of No Lateral Interactions

In the case when all possible membrane-mediated interactions of gA species and inclusions are absent, the partition function given by Equation (1) supplemented by Equations (2)–(5) can be calculated explicitly:

(12) $$Z^{id}=S^{m}\frac{1}{2^{N_{1}+N_{2}}}\frac{1}{N_{1}!N_{2}!}{\left(-Z_{\mathrm{int}}e^{-\frac{W_{D}}{T}}\right)}^{N_{1}}{\left(\frac{ST}{\pi M\hbar^{2}}\right)}^{N_{2}}U\left(-N_{1},1-N_{1}+N_{2},-\frac{ST}{\pi M\hbar^{2}Z_{\mathrm{int}}}e^{-\frac{W_{D}}{T}}\right),$$

where *U* is the Tricomi’s (confluent hypergeometric) function. Formally, the obtained partition function Equation (12) allows solving the problem of determination of the average number of dimers. In particular, in the limit of the large area, it yields the well-known relation for the dimer concentration [27]:

(13) $$C_{D}^{id}=D_{0}\left(C_{1}-C_{D}^{id}\right)\left(C_{2}-C_{D}^{id}\right),$$

where $C_{D}^{id}$ is the concentration of dimers in the ideal case of absent interactions; *D*_0_ is the monomer-dimer equilibrium constant in this case. This relation directly follows from Equation (12):

(14) $$k_{0}=C_{D}^{id}S=\frac{-T\partial \ln Z^{id}}{\partial W_{D}}=\frac{e^{-\frac{W_{D}}{T}}N_{U}N_{L}}{S}Z_{\mathrm{int}}\frac{\pi M\hbar^{2}}{T}=\frac{e^{-\frac{W_{D}}{T}}\left(N_{1}-k_{0}\right)\left(N_{2}-k_{0}\right)}{S}Z_{\mathrm{int}}\frac{\pi M\hbar^{2}}{T}.$$

A similar relation can be analytically obtained directly from the partition function, Equations (1)–(5), by the saddle point method. Let’s rewrite Equation (5) using the Stirling’s formula:

(15) $$\begin{array}{cc} Z & =const\cdot \sum_{k=0}^{\min(N_{1},N_{2})}e^{-k\left(1+\frac{W_{D}}{T}\right)}k^{-\frac{1}{2}-k}{\left(-k+N_{1}\right)}^{-\frac{1}{2}+k-N_{1}}{\left(-k+N_{2}\right)}^{-\frac{1}{2}+k-N_{2}}{\left(\frac{\pi \hbar^{2}M}{ST}Z_{\mathrm{int}}\right)}^{k}\\ & =const\sum_{k=0}^{\min\left(N_{1},N_{2}\right)}e^{f\left(k\right)}\approx const\int_{0}^{\min\left(N_{1},N_{2}\right)}e^{f(k)}dk, \end{array}$$

where *const* is some function independent on the number of dimers, *k*; *f*(*k*) is the logarithm of the expression standing under the summation sign in the upper line of the equation. The function *f*(*k*) has a maximum, which can be obtained from the equation:

(16) $$\frac{\partial f}{\partial k}=-\frac{W_{D}}{T}+\ln\left(\frac{\pi \hbar^{2}M}{T}Z_{\mathrm{int}}\right)+\ln C_{U}+\ln C_{L}-\ln C_{D}+O\left(1/k\right)=0.$$

From this expression it is seen that in the limit of large number of particles, the maximum of *f*(*k*) is achieved at *k* = *k*_0_, where *k*_0_ is determined by Equation (14). In the vicinity of the maximum:

(17) $$\begin{array}{l} f(k)\approx f(k_{0})-\frac{1}{2}\left[\frac{1}{N_{1}-k_{0}}+\frac{1}{N_{2}-k_{0}}+\frac{1}{k_{0}}-\frac{1}{2}\frac{1}{{\left(N_{1}-k_{0}\right)}^{2}}-\frac{1}{2}\frac{1}{{\left(N_{2}-k_{0}\right)}^{2}}-\frac{1}{2}\frac{1}{k_{0}^{2}}\right]{\left(k-k_{0}\right)}^{2}\\ \approx f(k_{0})-\frac{1}{2}\left[\frac{1}{N_{1}-k_{0}}+\frac{1}{N_{2}-k_{0}}+\frac{1}{k_{0}}\right]{\left(k-k_{0}\right)}^{2}. \end{array}$$

As *N*_1_, *N*_2_, *k*_0_ are much larger than 1, the integral in Equation (15) is majorly accumulated in the vicinity of *k*_0_. This allows rewriting Equation (15) as:

(18) $$Z=const\cdot \int_{-\infty }^{+\infty }e^{f(k)}dk=const\cdot \sqrt{\frac{2\pi }{\left(\frac{1}{N_{1}-k_{0}}+\frac{1}{N_{2}-k_{0}}+\frac{1}{k_{0}}\right)}}\exp\left[f\left(k_{0}\right)\right].$$

This partition function yields the same average number of dimers $\langle k\rangle =k_{0}$, as the partition function given by Equation (12). The square root in Equation (18) can be omitted, as the average number of dimers in the macroscopic limit is insensitive to this factor.

### 4.3. Equilibrium between gA Monomers and Dimers: The First Order of Interactions

Let’s consider the partition function given by Equations (1)–(5) in the linear order on the number of dimers. In this case, the polynomial *P*(*k*), Equation (11), should be truncated on linear term on *k*: *P* = *p*_0_ + *α*_1_*k*, where *p*_0_ and *α*_1_ are independent on *k*, although they can depend on *N*_1_, *N*_2_, *m*, *T*. Then the partition function can be expressed as (compare to Equation (12)):

(19) $$\begin{array}{l} Z=e^{p_{0}}S^{m}\frac{1}{2^{N_{1}+N_{2}}}\frac{1}{N_{1}!N_{2}!}{\left(-Z_{\mathrm{int}}e^{-\frac{W_{D}^{*}}{T}}\right)}^{N_{1}}{\left(\frac{ST}{\pi M\hbar^{2}}\right)}^{N_{2}}\\ \times U\left(-N_{1},1-N_{1}+N_{2},-\frac{ST}{\pi M\hbar^{2}Z_{\mathrm{int}}}e^{-\frac{W_{D}^{*}}{T}}\right), \end{array}$$

where $W_{D}^{*}$/*T* = *W*_D_/*T* − *α*_1_. Analogously to Equation (14), the average number *k*_1_ of dimers in the first-order approximation can be obtained as:

(20) $$k_{1}=\frac{e^{-\frac{W_{D}}{T}+\alpha_{1}}\left(N_{1}-k_{1}\right)\left(N_{2}-k_{1}\right)}{S}Z_{\mathrm{int}}\frac{\pi M\hbar^{2}}{T}=D_{0}\frac{e^{\alpha_{1}}\left(N_{1}-k_{1}\right)\left(N_{2}-k_{1}\right)}{S},$$

and, consequently, the concentration of dimers in the first-order approximation:

(21) $$C_{D}^{1st}=D_{0}e^{\alpha_{1}}\left(C_{1}-C_{D}^{1st}\right)\left(C_{2}-C_{D}^{1st}\right),$$

where *D*_0_ is the monomer-dimer equilibrium constant in the limit of low concentrations; the same *D*_0_ stands in Equation (13).

### 4.4. Equilibrium between gA Monomers and Dimers: Higher-Order Terms

Below, we obtain the average number of dimers taking into account *k*^2^ and higher order terms in Equation (11). Up to the fourth order, one can present the polynomial *P*(*k*) = *p*_0_ + *α*_1_*k* + *α*_2_*k*^2^ + *α*_3_*k*^3^ + *α*_4_*k*^4^, where *α*_1_, *α*_2_, *α*_3_, *α*_4_ are independent on *k*, although they can depend on *N*_1_, *N*_2_, *m*, *T*. In this case, Equation (16) yields:

(22) $$\frac{\partial f}{\partial k}=-\frac{W_{D}^{*}}{T}+\ln\left(\frac{\pi \hbar^{2}M}{T}Z_{\mathrm{int}}\right)+\ln C_{U}+\ln C_{L}-\ln C_{D}+2\alpha_{2}C_{D}+3\alpha_{3}C_{D}^{2}+4\alpha_{4}C_{D}^{3}=0.$$

This equation cannot be solved analytically. To obtain the approximate solution, we utilize the perturbation theory. We assume that the average equilibrium concentration of dimers obtained taking into account *k*^2^ and higher order terms in Equation (11), differ but slightly from the equilibrium concentration obtained in the first-order approximation as given by Equation (21), i.e., that for $\Delta C_{D}=C_{D}-C_{D}^{1st}$, the relation $\left|\Delta C_{D}\right|\ll C_{D}^{1st}$ holds. The Taylor series of Equation (22) up to the third order on *C*_D_ yields:

(23) $$\begin{array}{l} \frac{\partial f}{\partial k}=\ln\left(1-\frac{\Delta C_{D}}{C_{1}-C_{D}^{1st}}\right)+\ln\left(1-\frac{\Delta C_{D}}{C_{2}-C_{D}^{1st}}\right)-\ln\left(1+\frac{\Delta C_{D}}{C_{D}^{1st}}\right)+2\alpha_{2}C_{D}+3\alpha_{3}C_{D}^{2}+4\alpha_{4}C_{D}^{3}\\ =-[\frac{\Delta C_{D}}{C_{1}-C_{D}^{1st}}+\frac{1}{2}{\left(\frac{\Delta C_{D}}{C_{1}-C_{D}^{1st}}\right)}^{2}+\frac{1}{3}{\left(\frac{\Delta C_{D}}{C_{1}-C_{D}^{1st}}\right)}^{3}+\frac{\Delta C_{D}}{C_{2}-C_{D}^{1st}}+\frac{1}{2}{\left(\frac{\Delta C_{D}}{C_{2}-C_{D}^{1st}}\right)}^{2}\\ +\frac{1}{3}{\left(\frac{\Delta C_{D}}{C_{2}-C_{D}^{1st}}\right)}^{3}]-\frac{\Delta C_{D}}{C_{D}^{1st}}+\frac{1}{2}{\left(\frac{\Delta C_{D}}{C_{D}^{1st}}\right)}^{2}-\frac{1}{3}{\left(\frac{\Delta C_{D}}{C_{D}^{1st}}\right)}^{3}+2\alpha_{2}\left(C_{D}^{1st}+\Delta C_{D}\right)\\ +3\alpha_{3}{\left(C_{D}^{1st}+\Delta C_{D}\right)}^{2}+4\alpha_{4}{\left(C_{D}^{1st}+\Delta C_{D}\right)}^{3}=0. \end{array}$$

Further, we take Taylor series of *C*_D_ with respect to $C_{D}^{1st}$ up to the fourth order, substitute *C*_D_ into Equation (23) and under the assumptions $C_{D}^{1st}\ll C_{1},C_{2}$ express the coefficients of the Taylor series as:

(24) $$\begin{array}{cc} \Delta C_{D} & =2\alpha_{2}{\left(C_{D}^{1st}\right)}^{2}+\left(3\alpha_{3}+6\alpha_{2}^{2}-2\alpha_{2}\left(\frac{1}{C_{1}}+\frac{1}{C_{2}}\right)\right){\left(C_{D}^{1st}\right)}^{3}\\ & +\left(4\alpha_{4}+24\alpha_{2}\alpha_{3}+\frac{64}{3}\alpha_{2}^{3}-\left(3\alpha_{3}+14\alpha_{2}^{2}\right)\left(\frac{1}{C_{1}}+\frac{1}{C_{2}}\right)+4\alpha_{2}\frac{1}{C_{1}C_{2}}\right){\left(C_{D}^{1st}\right)}^{4}. \end{array}$$

The series given by Equation (23) is formally correct when $\alpha_{2}C_{\dim0}\ll 1$, $\alpha_{3}C_{\dim0}^{2}\ll 1$, $\alpha_{4}C_{\dim0}^{3}\ll 1$.

Figure 2 and Figure 3 demonstrate that all particles (U, L, D, I) on the average attract to transmembrane inclusions (gA dimers and transmembrane peptides), that results in positive values of corresponding Mayer cluster integrals *β*_1,AB_, *β*_2,ABC_, *β*_3,ABCE_. The interaction energies are negative and their absolute values are quite large, of the order of several *k_B_T*. This allows assuming qualitatively, that the main contribution to the shift of monomer-dimer equilibrium comes from interactions involving transmembrane inclusions, and thus all coefficients *α_i_* should be positive. Consequently, we obtain the lower estimate for the concentrations of dimers.

### 4.5. Lifetime of Dimers

Consider the gA dimer surrounded by gA monomers, other gA dimers, and membrane inclusions. The inverse average lifetime of the dimer, 1/*τ*, is determined by the product of the Boltzmann factor exp[–Δ*W*/*T*] (where Δ*W* is the energy barrier of the dimer dissociation) and the frequency of attempts, *ν*, to dissociate:

(25) $$\frac{1}{\tau }=\nu \exp\left[\frac{-\Delta W}{T}\right].$$

Following [34,35,36], we assume that the configuration of the coaxial pair of gA monomers corresponds to the top of the energy barrier. In this configuration, two gA monomers from opposing membrane leaflets stand one on top of the other and do not form the dimer. Thus, the energy barrier *W* is given by the difference of the energy of the pair and dimer configurations. The presence of gA monomers, gA dimers, and membrane inclusions influences the values of these two energies, and, consequently, alters the average lifetime of the dimer. In particular, in the works [43,44,45] it was demonstrated that the lifetime of tandem gA channels formed from two lateral dimers of gA, exceeds the lifetime of regular gA channels by about three orders of magnitude.

In order to obtain the inverse average lifetime of dimers in the membrane, one should average the expression Equation (25) with respect to positions of all particles present in the membrane:

(26) $$\begin{array}{ll} \frac{1}{\tau } & =\langle \nu \exp\left[\frac{-\Delta W}{T}\right]\rangle =\nu \frac{\int \exp\left[-\frac{W_{P,all}-W_{D,all}}{T}\right]\exp\left[-\frac{W_{D,all}}{T}\right]\Pi dr_{i}}{\int \exp\left[-\frac{W_{D,all}}{T}\right]\Pi dr_{i}}\\ & =\nu \frac{\int \exp\left[-\frac{W_{P,all}}{T}\right]\Pi dr_{i}}{\int \exp\left[-\frac{W_{D,all}}{T}\right]\Pi dr_{i}}, \end{array}$$

where *W*_P,*all*_ is the energy of the system comprising gA monomers, dimers and inclusions supplemented by single coaxial pair of gA monomers; *W*_D,*all*_ is the energy of the system comprising gA monomers, dimers and inclusions supplemented by single dimer (below, such dimer is denoted as D′). Actually, the relation (26) can be considered as a ratio of two partition functions. Utilizing the results on the formal structure of the partition functions obtained in the previous subsections, we can express 1/*τ* as follows:

(27) $$\frac{1}{\tau }=\frac{1}{\tau_{0}}\frac{\int \exp\left[K_{1}+K_{2}+K_{3}+\ldots \right]\rho \left(k\right)dk}{\int \exp\left[H_{1}+H_{2}+H_{3}+\ldots \right]\rho \left(k\right)dk},$$

where *τ*_0_ is the lifetime of single solitary dimer; *K*_1_, *K*_2_, *K*_3_ are contributions from diagrams (Figure 1) that include exactly one coaxial pair; *H*_1_, *H*_2_, *H*_3_ are contributions from diagrams (Figure 1) that include at least one dimer; *ρ*(*k*) = exp[*f*(*k*)] is the distribution function of the number of dimers. Below we present the explicit expressions for *K*_1_, *H*_1_, *K*_2_; other contributions can be obtained in analogous way:

(28) $$\begin{array}{cc} K_{1} & =\beta_{1,\mathrm{PU}}C_{U}+\beta_{1,\mathrm{PL}}C_{L}+\beta_{1,\mathrm{PD}}C_{D}+\beta_{1,\mathrm{PI}}C_{I},\\ H_{1} & =\beta_{1,D^{\prime }U}C_{U}+\beta_{1,D^{\prime }L}C_{L}+\beta_{1,D^{\prime }D}C_{D}+\beta_{1,D^{\prime }I}C_{I},\\ K_{2} & =\beta_{2,\mathrm{PUU}}\frac{C_{U}^{2}}{2}+\beta_{2,\mathrm{PUL}}C_{U}C_{L}+\beta_{2,\mathrm{PLL}}\frac{C_{L}^{2}}{2}\\ & +\beta_{2,\mathrm{PUD}}C_{U}C_{D}+\beta_{2,\mathrm{PLD}}C_{L}C_{D}+\beta_{2,\mathrm{PDD}}\frac{C_{D}^{2}}{2}\\ & +\beta_{2,\mathrm{PUI}}C_{U}C_{I}+\beta_{2,\mathrm{PLI}}C_{L}C_{I}+\beta_{2,\mathrm{PDI}}C_{D}C_{I}+\beta_{2,\mathrm{PII}}\frac{C_{I}^{2}}{2}. \end{array}$$

Actually, it is not necessary to explicitly calculate the integrals standing in Equation (27), as in the macroscopic limit the corresponding integrals generally accumulate in the vicinity of the average value k=〈k〉, i.e., the distribution function *ρ*(*k*) is very narrow (close to the corresponding *δ*-function), and thus:

(29) $$\frac{1}{\tau }\approx \frac{1}{\tau_{0}}\frac{\exp\left[K_{1}+K_{2}+K_{3}+\ldots \right]}{\exp\left[H_{1}+H_{2}+H_{3}+\ldots \right]}=\frac{1}{\tau_{0}}\exp[\left(K_{1}-H_{1}\right)+\left(K_{2}-H_{2}\right)+\left(K_{3}-H_{3}\right)+\ldots ].$$

In this equation, the correction to the dimer concentration calculated in accordance with Equation (24) should be taken into account. In the case of absence of membrane inclusions, one can explicitly check that in the first order on concentrations *C*_1_, *C*_2_ from Equation (29) it follows:

(30) $$\tau =\tau_{0}\left(1+\left(\beta_{1,\mathrm{UD}}-\beta_{1,\mathrm{PU}}\right)C_{1}+\left(\beta_{1,\mathrm{LD}}-\beta_{1,\mathrm{PL}}\right)C_{2}\right)+o\left(C\right),$$

That coincides with the results obtained in the work [36]. Thus, in the regime of low concentrations, the main contribution to the lifetime correction is provided from interactions of monomers with pairs and dimers. Increasing concentrations lead to growing contributions from dimer-dimer and dimer-pair interactions. By considering the limit *C*_1_, *C*_2_ → 0, one can obtain from Equation (29) the dependence of the lifetime of the solitary dimer on the concentration of membrane inclusions:

(31) $$\begin{array}{cc} \tau & =\tau_{0}\exp[\left(\beta_{1,\mathrm{DI}}-\beta_{1,\mathrm{PI}}\right)C_{I}+\left(\beta_{2,\mathrm{DII}}-\beta_{2,\mathrm{PII}}\right)\frac{C_{I}^{2}}{2!}\\ & +\left(\beta_{3,\mathrm{DIII}}-\beta_{3,\mathrm{PIII}}\right)\frac{C_{I}^{3}}{3!}]. \end{array}$$

### 4.6. Calculation of Interaction Energies Based on Theory of Elasticity of Lipid Membranes

We calculate the energy of membrane-mediated interaction of gA monomers, dimers, pairs and membrane inclusions based on the theory of elasticity of lipid membranes. Each the particle induces elastic deformations in its vicinity. When the particles are far separated, their induced deformations are independent and their energies are additive. Upon mutual approach, the deformations overlap leading to effective lateral interaction between the particles. The method of calculation of the interaction energies is described in details in [34,35,36] for gA monomers and dimers, and in [20]—for amphipathic peptides. Here we briefly outline the algorithm of the calculation.

We introduce a Cartesian coordinate system *Oxyz* in such a way that the axis *Oz* is directed perpendicular to the plane of the undisturbed lipid bilayer; the coordinate origin *O* is placed at the monolayer interface. The upper monolayer lies in the half-space *z* > 0, the lower monolayer—*z* < 0. We describe elastic deformations of the lipid membrane considering it as a continuous elastic medium. The deformations are deemed small and their energy is calculated in quadratic order, analogously to the approach developed by Hamm and Kozlov [54]. The shapes of the upper and lower monolayer surfaces are characterized by *z*-coordinates of their points, described by functions *H_u_*(*x*, *y*) and *H_l_*(*x*, *y*), respectively. We describe *z*-coordinates of the monolayer interface by the function *M*(*x*, *y*). The average orientation of lipid molecules in the upper and lower monolayers is described by unit vectors **n***_u_* and **n***_l_*, respectively, called directors. Along with functions *H_u_*(*x*, *y*) and *H_l_*(*x*, *y*), the shapes of the surfaces of the upper and lower monolayers are determined by unit normal vectors **N***_u_* and **N***_l_*, respectively. We account for the following deformations: splay characterized by inhomogeneity of the director; tilt characterized by deviation of directors from normal to monolayer surface, **t** = **n**/(**nN**) − **N** ≈ **n** − **N**; lateral stretching characterized by relative change of the area of the monolayer surface *α* = (*a* − *a*_0_)/*a*_0_, (*a*, *a*_0_ are current and initial area per lipid molecule, respectively); lateral tension *σ*; the Gaussian splay $K=\frac{\partial n_{x}}{\partial x}\frac{\partial n_{y}}{\partial y}-\frac{\partial n_{x}}{\partial y}\frac{\partial n_{y}}{\partial x}$; and the deformation of twist characterized by **rot**(**n**). The energy of elastic deformations of the lipid monolayer can be written as [20,34,35,36]:

(32) $$\begin{array}{cc} W & =\int dS(\frac{B}{2}{\left(\mathrm{div}\left(n\right)+J_{0}\right)}^{2}-\frac{B}{2}J_{0}^{2}+\frac{K_{t}}{2}t^{2}+\frac{\sigma }{2}{\left(grad\;\left(H\right)\right)}^{2}\\ & +\frac{K_{a}}{2}\alpha^{2}+K_{G}K+\frac{K_{\mathrm{rot}}}{2}{\left(rot\;\left(n\right)\right)}^{2}), \end{array}$$

where *B*, *K_t_*, *K_a_*, *K_G_*, *K*_rot_ are moduli of splay, tilt, lateral stretching, Gaussian splay, twist, respectively; *J*_0_ is monolayer spontaneous curvature; the integration is performed over the monolayer surface. We impose the condition of local volumetric incompressibility onto the monolayer, as the bulk modulus of the membrane is very large (~10^4^ *k_B_T*/nm^3^ [59]). In the case of small deformations, this condition yields:

(33) $$H_{u}-M=h-\frac{h^{2}}{2}\mathrm{div}\left(n_{u}\right)-h\alpha_{u},\;M-H_{l}=h-\frac{h^{2}}{2}\mathrm{div}\left(n_{l}\right)-h\alpha_{l},$$

where *h* is the thickness of the hydrophobic part of the lipid monolayer. Within the required accuracy, $n_{u,l}={\left(n_{u,l}{}_{x}\left(x,y\right),n_{u,l}{}_{y}\left(x,y\right),\mp 1\right)}^{T}$, $N_{u,l}={\left(\pm \partial H_{u,l}\left(x,y\right)/\partial x,\pm \partial H_{u,l}\left(x,y\right)/\partial y,\mp 1\right)}^{T}$, where the upper signs correspond to the upper monolayer, the lower signs—to the lower monolayer; *T* in superscript is the transposition, subscripts “*x*”, “*y*” denote the projection onto the corresponding axis. Using Equation (33), one can rewrite Equation (32) as follows:

(34) $$\begin{array}{ll} W & =\int dS_{u}(\frac{B}{2}{\left(\mathrm{div}\left(n_{u}\right)+J_{u}\right)}^{2}-\frac{B}{2}J_{u}^{2}+\frac{K_{t}}{2}{\left(n_{u}-grad\;\left(H_{u}\right)\right)}^{2}+\frac{\sigma_{u}}{2}{\left(grad\;\left(H_{u}\right)\right)}^{2}\\ & +\frac{K_{a}}{2h^{2}}{\left(h-\frac{h^{2}}{2}\mathrm{div}\left(n_{u}\right)+M-H_{u}\right)}^{2}+K_{G}K_{u}+\frac{K_{\mathrm{rot}}}{2}{\left(rot\;\left(n_{u}\right)\right)}^{2})\\ & +\int dS_{l}(\frac{B}{2}{\left(\mathrm{div}\left(n_{l}\right)+J_{l}\right)}^{2}-\frac{B}{2}J_{l}^{2}+\frac{K_{t}}{2}{\left(n_{l}+grad\;\left(H_{l}\right)\right)}^{2}+\frac{\sigma_{l}}{2}{\left(grad\;\left(H_{l}\right)\right)}^{2}\\ & +\frac{K_{a}}{2h^{2}}{\left(h-\frac{h^{2}}{2}\mathrm{div}\left(n_{l}\right)-M+H_{l}\right)}^{2}+K_{G}K_{l}+\frac{K_{\mathrm{rot}}}{2}{\left(rot\;\left(n_{l}\right)\right)}^{2}). \end{array}$$

The energy functional Equation (34) should be supplemented by boundary conditions. The membrane is assumed to be undeformed far from the membrane-deforming particles, i.e.,

(35) $$n_{u,l}\left(\infty \right)={\left(0,0,\mp 1\right)}^{T},\;M\left(\infty \right)=0,\;H_{u}\left(\infty \right)=h,\;H_{l}\left(\infty \right)=-h.$$

Besides, specific boundary conditions are imposed at the boundary of membrane-deforming particles. Let’s denote the outline of the gA monomer, dimer, or pair at the monolayer surface as Γ. Γ is assumed to be a circle of the radius *r*_0_, the center of which lies at the axis of rotational symmetry of the gA monomer, dimer or pair. We suppose that gA dimer imposes the following boundary conditions at the circle:

(36) $$H_{u}\left(\Gamma \right)=H_{0}+h_{p},\;H_{l}\left(\Gamma \right)=H_{0}-h_{p},$$

where *h_p_* is the length of the gA monomer along the membrane normal; *H*_0_ is the *z*-coordinate of the dimer center, determined from the minimization of the energy functional Equation (34) subjected to the boundary conditions Equations (35) and (36). The monomer and pair are supposed to impose boundary conditions on the director. For vertical gA monomer located in the upper monolayer the boundary conditions read:

(37) $$n_{u,n}\left(\Gamma \right)=-\frac{h-h_{p}}{\sqrt{{\left(h-h_{p}\right)}^{2}+h_{p}^{2}}},\;n_{u,t}\left(\Gamma \right)=0,\;H_{u}\left(\Gamma \right)=H_{m},$$

where *n_u,n_* and *n_u,t_* are normal and tangential components of the director with respect to the boundary Γ; *H_m_* is *z*-coordinate of the circle Γ determined from the minimization of the energy functional Equation (34). If the monomer is tilted with respect to the vertical axis, then the boundary conditions Equation (37) are modified as follows:

(38) $$n_{u}\left(\Gamma \right)=n_{0}\left(\Gamma \right)+\Delta n,\;H_{u}\left(\Gamma \right)=H_{m}+\Delta r\Delta n,$$

where **n**_0_ is the boundary director of vertically standing monomer, Equation (37); Δ**n** is the vector characterizing the tilt of the monomer determined from the minimization of the elastic energy functional, Equation (34); Δ**r** = **r** − **r**_0_, where **r**_0_ is the radius-vector of the center of the circle Γ and **r** is the radius-vector of a point on the contour Γ. For gA monomer located in the lower monolayer the boundary conditions are analogous. For the coaxial pair the same boundary conditions should be imposed simultaneously for the upper and lower monomer of the pair.

As the transmembrane inclusion, we consider a cylindrically shaped transmembrane peptide, the radius of which coincides with the radius of the gramicidin dimer, and the length of which is smaller than the hydrophobic thickness of the lipid bilayer. The membrane has to compress in the vicinity of such inclusions in order to avoid an exposure of hydrophobic membrane interior to polar media (either water or polar lipid heads). We impose the following boundary conditions on the transmembrane inclusions:

(39) $$\begin{array}{l} H_{u}\left(\Gamma \right)=H_{I0}+h_{I},\\ H_{l}\left(\Gamma \right)=H_{I0}-h_{I},\\ n_{u,n}\left(\Gamma \right)=0,\;n_{u,\tau }\left(\Gamma \right)=0,\;n_{l,n}\left(\Gamma \right)=0,\;n_{l,\tau }\left(\Gamma \right)=0,\; \end{array}$$

where *h*_I_ is the half of the inclusion length; *H*_I0_ is analogous to *H*_0_ in Equation (36); it is determined from minimization of the energy functional, Equation (34).

Besides, we consider inactivated monomers of gramicidin as peripheral membrane inclusions. Such monomers are generated, e.g., in the course of photo-induced damage of gramicidin by reactive oxygen species generated by excited photosensitizers [36,60,61]. At the inactivated monomers, we impose the same boundary conditions, as at the intact gramicidin monomers; the only difference is that inactivated monomers cannot participate in the dimerization reaction. Inactivated monomers can also be considered as short amphipathic peptides, as their imposed boundary conditions coincide [36]. In the present work we do not consider long helical amphipathic peptides, as their strict analysis requires explicit accounting for their rotational degree of freedom in the plane of the membrane. Such consideration would substantially complicate the calculation of configuration partition functions. This problem is beyond the scope of the present study, although it principally can be solved in the framework of the developed formalism.

### 4.7. Parameters of the System

To obtain quantitative results, we utilized the following values of the membrane elastic parameters, typical for diphytanoylphosphatidylcholine (DPhPC): splay modulus *B* = 14 *k_B_T* (*k_B_T* ≈ 4 × 10^−21^ J) [62]; tilt modulus *K_t_* = 40 mN/m = 10 *k_B_T*/nm^2^ [54]; the modulus of lateral stretching-compression *K_a_* = 133 mN/m [63]; twist modulus *K*_rot_ = *B*/2 = 7 *k_B_T*; the modulus of the Gaussian splay *K_G_* = −*B*/2 = −7 *k_B_T* [64]; spontaneous curvature *J*_0_ = −0.015 nm^−1^; hydrophobic thickness *h* = 1.4 nm [42,63]; all values are related to the lipid monolayer. The length and the radius of the gramicidin monomer were *h_p_* = 0.75 nm and *r*_0_ = 1 nm, respectively [34,35,36]. The length and radius of the transmembrane inclusion were taken 2*h*_I_ = 1.7 nm and *r*_I_ = *r*_0_ = 1 nm, respectively.

### 4.8. Numerical Minimization of the Elastic Energy Functional

The functional of the membrane elastic energy given by Equation (34) was minimized numerically, as the minimization cannot be performed analytically under the imposed boundary conditions. We utilized the finite element method with an adaptive mesh. The functions in the functional Equation (34) were replaced by their piecewise linear interpolants on each triangle of the computational mesh, the size of which decreased while approaching the membrane inclusions. The calculation was performed for meshes of different average sizes, and further the result was extrapolated towards the zero size mesh. The procedure of the numerical minimization of the elastic energy functional Equation (34) is described in details in the works [20,34,35,36,65]. The resulting interaction potentials are presented in Figure 2 and Figure 3.

### 4.9. Numerical Calculation of the Mayer’s Cluster Integrals

The Mayer’s cluster integrals *β*_1,AB_, *β*_2,ABC_, *β*_3,ABCE_ standing as coefficients in the polynomial, Equation (11), were calculated according to diagrams (Figure 1) utilizing the Monte Carlo method. For configurations of overlapping monomers in one monolayer, overlapping dimers, etc. the energy was set at the value equal to +∞. In the region where the interaction energies are finite, all calculated interaction energies were approximated by the function *w*(*r*) = *e*^−*qr*^*P*_8_(*r*), where *q* is some fitting constant, and *P*_8_(*r*) is the approximation polynomial of the eighth degree. The deviation of the approximation from the explicitly calculated energies did not exceed 2%. The explicit values of the integrals *β*_1,AB_ are presented in Table 1; *β*_2,ABC_—in Table 2; *β*_3,ABCE_—in Table 3.

## Figures and Tables

**Table 1:** Values of integrals *β*_1,AB_ for different types of interacting particles. The values are in the units of nm^2^.

Type of Particle	U	L	D	I	P
**U**	–104	68.7	991	88.6	–15.8
**L**	68.7	–104	991	88.6	–15.8
**D**	991	991	101,659	12,701	59,529
**I**	88.6	88.6	12,701	9517	1636
**P**	–15.8	–15.8	59,529	1636	—

**Table 2:** Values of integrals *β*_2,ABC_ for different types of interacting particles. The values are in the units of nm^4^.

ABC	UUU LLL	UUL ULL	UUD LLD	ULD	UUI LLI	ULI	UUP LLP
*β*_2,ABC_	–2312	–1750	–476,256	–359,087	–3915	–3218	–2140
ABC	ULP	UDD LDD	UII LII	UDP LDP	UIP LIP	UDI LDI	**DDI**
*β*_2,ABC_	–2267	9.5 × 10^8^	1.1 × 10^6^	–2.7 × 10^7^	–37,800	1.2 × 10^7^	2.1 × 10^11^
ABC	DII	DDD	PDI	PII	III	PDD	
*β*_2,ABC_	2.0 × 10^11^	1.4 × 10^13^	1.1 × 10^10^	1.8 × 10^8^	1.1 × 10^10^	3.4 × 10^12^	

**Table 3:** Values of integrals *β*_3,ABCE_ for different types of interacting particles. The values are in the units of nm^6^.

ABCE	UUUU LLLL	UUUL LLLU	UULL	UUUD LLLD	UULD LLUD	UUUI LLLI	UULI LLUI
*β*_3,ABCE_	–39,320	–70,375	–27,268	5.4 × 10^8^	3.5 × 10^8^	320,627	36,887
ABCE	UUUP LLLP	UULP LLUP	UUDD LLDD	ULDD	UUDI LLDI	ULDI	UUII LLII
*β*_3,ABCE_	–162,519	–94,637	2.8 × 10^12^	4.3 × 10^12^	–9.5 × 10^9^	–5.6 × 10^9^	–1.5 × 10^8^
ABCE	ULII	UUDP LLDP	ULDP	UUIP LLIP	ULIP	UDDD LDDD	UDDI LDDI
*β*_3,ABCE_	–8.3 × 10^7^	2.9 × 10^10^	2.3 × 10^10^	–337,172	–2.0 × 10^6^	1.1 × 10^18^	3.8 × 10^15^
ABCE	UDII LDII	UIII LIII	UDDP LDDP	UDIP LDIP	UIIP LIIP	DDDD	DDDI
*β*_3,ABCE_	5.3 × 10^13^	3.7 × 10^12^	1.4 × 10^16^	–1.2 × 10^12^	5.3 × 10^9^	1.5 × 10^21^	1.0 × 10^19^
ABCE	DDII	DIII	IIII	DDDP	DDIP	DIIP	IIIP
*β*_3,ABCE_	2.4 × 10^17^	2.6 × 10^16^	1.5 × 10^16^	4.9 × 10^22^	9.0 × 10^19^	2.2 × 10^17^	1.5 × 10^15^
